# Characterization of novel regulators for heat stress tolerance in tomato from Indian sub‐continent

**DOI:** 10.1111/pbi.13371

**Published:** 2020-09-01

**Authors:** Sonia Balyan, Sombir Rao, Sarita Jha, Chandni Bansal, Jaishri Rubina Das, Saloni Mathur

**Affiliations:** ^1^ National Institute of Plant Genome Research New Delhi India

**Keywords:** heat, tomato cultivar, tolerant, CLN1621L, CA4, transcriptome

## Abstract

The footprint of tomato cultivation, a cool region crop that exhibits heat stress (HS) sensitivity, is increasing in the tropics/sub‐tropics. Knowledge of novel regulatory hot spots from varieties growing in the Indian sub‐continent climatic zones could be vital for developing HS‐resilient crops. Comparative transcriptome‐wide signatures of a tolerant (CLN1621L) and sensitive (CA4) cultivar pair shortlisted from a pool of varieties exhibiting variable thermo‐sensitivity using physiological‐, survival‐ and yield‐related traits revealed redundant to cultivar‐specific HS regulation. The antagonistically expressing genes encode enzymes and proteins that have roles in plant defence and abiotic stresses. Functional characterization of three antagonistic genes by overexpression and silencing established Solyc09g014280 (*Acylsugar acyltransferase*) and Solyc07g056570 (*Notabilis*) that are up‐regulated in tolerant cultivar, as positive regulators of HS tolerance and Solyc03g020030 (*Pin‐II proteinase inhibitor*), that are down‐regulated in CLN1621L, as negative regulator of thermotolerance. Transcriptional assessment of promoters of these genes by SNPs in stress‐responsive *cis*‐elements and promoter swapping experiments in opposite cultivar background showed inherent cultivar‐specific orchestration of transcription factors in regulating transcription. Moreover, overexpression of three ethylene response transcription factors (ERF.C1/F4/F5) also improved HS tolerance in tomato. This study identifies several novel HS tolerance genes and provides proof of their utility in tomato thermotolerance.

## Introduction

Global warming is exposing plants to unfavourable temperature fluctuations resulting in detrimental effects on crop productivity and an ultimate threat to biodiversity and food security (Battisti and Naylor, 2009; Challinor *et al.*, 2014). As per the 5th report of Intergovernmental Panel on Climate Change (IPCC), 0.8–4.8 °C rise in the global mean temperatures has been projected by the end of this century (IPCC AR5, Pachauri *et al*., 2014). High temperature affects the phenological development, photosynthesis, respiration, initiation, expansion and senescence of plant organs (Wang *et al.*, 2017). As sessile life forms, it is imperative for plants to evolve mechanisms to adapt to the thermal stress to minimize damage on their growth and reproduction. This requires dynamic reprogramming at the level of transcriptome, proteome, metabolome, lipidome and epigenome leading to the activation of complex heat stress response (HSR) (Mittler *et al.*, 2012). It is therefore necessary to have in‐depth understanding of the plant thermotolerance mechanisms at molecular level to achieve sustainable food production. The canonical HSR involves HS sensing by Phytochrome B (Jung *et al.*, 2016), the heat‐induced modulations at the level of membrane stability activating the calcium and lipid signalling (Saidi *et al.*, 2010), and the interplay of conserved heat stress transcription factor‐heat shock protein (HSF‐HSP) module. Upon HS, HSFA1s, the master regulators of HSR, are relieved from repression by HSP70/90, relocate from cytoplasm to nucleus and activate other HSFs (HSFA7s, HSFA2 and HSFBs), dehydration‐responsive element‐binding protein 2A (DREB2A) and multiprotein bridging factor 1C (MBF1C) (Liu *et al.*, 2011; Mishra *et al.*, 2002; Yoshida *et al.*, 2011). These transcriptional regulators further control the expression of several HS‐inducible genes including HSFA3, nuclear factor‐Y (NF‐Y) and HSPs (Chen *et al.*, 2010; Sato *et al.*, 2014; Schramm *et al.*, 2008; Yoshida *et al.*, 2008, 2011). In Arabidopsis, HSFA1 regulates HSFBs that in turn finely regulate the HSR by repressing the activity of HSFA1s in a negative feedback loop (Ikeda *et al.*, 2011). Another critical regulator of thermotolerance is the C‐REPEAT BINDING FACTOR2 (RCF2)‐regulated NAC019 that acts as an upstream regulator of HSFA1b, HSFA6b, HSFA7a and HSFC1 (Guan *et al.*, 2014). Several HSF‐target modules like HSFA4a‐APX1 and HSFA5‐HSPs act independent of HSFA1 pathway (Liu *et al.*, 2006; Von Koskull‐Doring *et al.*, 2007). In addition, a flexible mode of heat response pathways involving bZIP28‐, HSFA2‐ and ROS‐dependent signals is also demonstrated in Arabidopsis (Kataoka *et al.*, 2017). Apart from these canonical HSR circuits, PIF4 regulatory and associated signalling networks control plant thermo‐morphogenesis (Koini *et al.*, 2009; Oh *et al.*, 2012; Proveniers and Van Zanten, 2013; Stavang *et al.*, 2009). The significance of post‐translational regulation in HS response is also well documented in plant HSR (Agarwal *et al.*, 2007; Hahn *et al.*, 2011; Miller *et al.*, 2010; Mizoi *et al.*, 2013; Sato *et al.*, 2016; Vainonen *et al.*, 2012). Evidences also suggest orchestration of multi‐layered regulatory systems comprising not only TFs but also epigenetic regulators and small RNAs as important HSR players. In Arabidopsis, methylation of histone H3 lysine 4 functions as modulator of HS memory for survival under recurring HS conditions by HSFA2‐mediated recruitment of histone methyltransferases (Lämke *et al.*, 2016).

Tomato (*Solanum lycopersicum)* is the second most cultivated vegetable in the world. HS leads to reduced tomato yield by affecting its vegetative as well as reproductive development (Pressman *et al.*, 2002; Sato *et al.*, 2000). Transcript profiling using cDNA‐AFLP (Bita *et al.*, 2011) and microarray analysis (Frank *et al.*, 2009) as well as proteomic analyses (Mazzeo *et al.*, 2018) of microspores has shown active involvement of HSPs, ROS scavengers, hormones, amino acid metabolism and nitrogen assimilation in tomato HS response. Heat‐inducible tomato HSP21 protects PSII from the HS‐induced oxidative stress and also plays role in fruit ripening (Neta‐Sharir *et al.*, 2005). Tomato HSFA2 is highly up‐regulated during HS that in turn forms a hetero‐oligomeric transcriptional complex with HSFA1. In addition, HSP17.4‐CII acts as a co‐repressor of HSFA2 (Port *et al.*, 2004). Overexpression of Arabidopsis receptor‐like kinase ERECTA (ER) leads to enhanced HS tolerance in Arabidopsis, rice and tomato with increased biomass (Shen *et al.*, 2015). The microRNA 169:NF‐Y A module has also been shown to be differentially regulated during different heat stress regimes in tomato (Rao *et al.*, 2020). Till now, the knowledge about the effect of HS on tomato leaf by exploiting the comparative transcriptomics in contrasting cultivars is not well understood. Tomato is increasingly becoming a cash crop in regions of higher temperatures (the tropics and sub‐tropics) than the optimum 26/20 °C during the photoperiod/dark period (Srivastava *et al.*, 2012). One way for developing HS‐resilient crops is to exploit the naturally evolved mechanistic signalling frameworks in cultivars exhibiting contrasting response to HS (Challinor *et al.*, 2014). To uncover the HS‐responsive molecular mechanisms in tomato using naturally occurring cultivars growing in the sub‐tropical climatic conditions of the Indian sub‐continent, we first evaluated nine tolerant/sensitive tomato cultivars under HS to identify the best contrasting pair, viz. CLN1621L (henceforth referred to as CLN, tolerant) and CA4 (sensitive). Comparative analysis of the heat‐responsive transcriptomic signatures of leaves highlighted conserved to genotype‐specific reprogramming at transcriptional levels. Functional assessment by silencing (virus‐induced gene silencing, VIGS) and transient overexpression of three previously uncharacterized genes, that exhibit antagonistic expression in the contrasting cultivars under HS, established their roles as positive/negative regulators of HS tolerance. Promoter:reporter expression analysis highlights that cultivar‐dependent transcription factors govern the opposite expression of these genes. The study provides a collection of canonical to cultivar‐biased HS‐responsive genes for genetic engineering of tomato for thermotolerance.

## Results and discussion

### Identification of HS‐tolerant and HS‐sensitive contrasting cultivar pair

**Figure 1:**
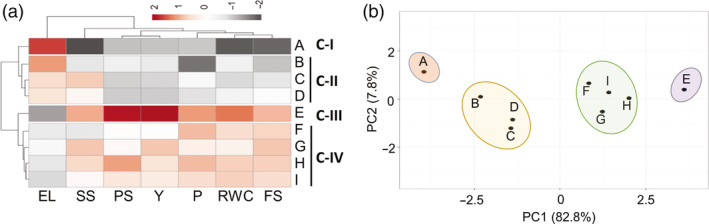
Identification of heat stress‐tolerant and sensitive tomato cultivars. (a) Nine tomato cultivars with variable heat stress tolerance were screened to select best contrasting pair to heat stress response by assessing physiological‐, biochemical‐ and yield‐related traits. Based on the performance of each cultivar, the percentage value of each trait was calculated under heat stress relative to respective controls which was set as 100%. The heat map generated using the above data for all the cultivars divided them into four classes I to IV. For heat map, a *Z*‐score scale (unit variance scaling was applied to rows) following Manhattan distance and complete linkage was used. (b) Principal component analysis of the nine cultivars on above parameters was done using ClustVis following singular value decomposition (SVD) with imputation to calculate principal components. Cultivars are represented by alphabets A to I. A: CA4; B: Pusa Ruby; C: Pusa‐120; D: Pusa Rohini; E: CLN1621L; F: Hisar Arun; G: IIHR2274; H: Pusa Sadabahar; I: IIHR220. EL, Electrolytic Leakage; SS, 5‐day‐old seedling survival; PS, 1‐month‐old plant survival; Y, Yield; P, proline content; RWC, relative water content; FS, percentage fruit set/plant.

The genomic variations between different cultivars within the same species are often armed with specific mechanisms to tolerate stress at morphological, physiological and molecular levels (Huang and Gao, 1999). Tomato production has considerably increased in the tropical and sub‐tropical regions (Nicola *et al.*, 2009; Srivastava *et al.*, 2012). To exploit the variability in thermotolerance, 9 tomato cultivars from Indian sub‐continent were screened and graded as tolerant and sensitive to shortlist best contrasting pair. These cultivars included five tolerant [CLN, IIHR2201, IIHR2274, Pusa Sadabahar and Hisar Arun] and four sensitive cultivars (CA4, Pusa Ruby, Pusa Rohini and Pusa120) (Chavan *et al.*, 2009; Kartikeya *et al.*, 2012; Meena and Bahadur, 2015; Sangu *et al.*, 2015). The tolerance of cultivars to HS was judged based on the parameters adopted by several previous reports for screening tomato cultivars under HS (Abdul‐Baki, 1991; Abdul‐Baki and Stommel, 1995). These include survival assays at seedling stage (Figure S1a) and at 1‐month‐old stage (Figure S1b); physiological assays like proline content, relative water content (RWC) and electrolytic leakage (EL) (Figure S1c–e); and yield‐related traits like percentage of fruit set per plant and total fruit yield (Figure S1f,g). Higher values for all the above parameters and lower values for EL are hallmarks of better thermotolerance. The cultivars were ranked for their performance by assaying all the above parameters as percentage increase/decrease under HS relative to control conditions. CLN displayed highest ranking among the cultivars for all the above parameters except EL (Figure 1a and Figure S1e). CA4 on the other hand had highest EL and obtained lowest rank for all other parameters except proline content where Pusa Ruby had least value (Figure 1a; Figure S1c). Of note, percentage survival of 1‐month‐old CLN plants was more than 80%, whereas it was mere 11% in CA4. Fruit set was also higher in CLN (81%) as compared to CA4 (27%), as reflected by nearly 50% yield being maintained in CLN but only 6% in CA4 (Figure S1f,g). Hierarchical clustering clearly divided the cultivars into four clades based on their HS response, viz. (i) most tolerant (CLN), (ii) moderately tolerant (Pusa Sadabahar, IIHR2274, Hisar Arun and IIHR2201), (iii) sensitive (Pusa Ruby, Pusa‐120 and Pusa Rohini) and (iv) highly sensitive (CA4) (Figure 1a). This was further strengthened by PCA of all the above factors that showed the separation of each cultivar on PC1 (82.8%) based on the performance under HS, placing CLN and CA4 on the two extremities (Figure 1b), thus identifying them as the most tolerant and sensitive cultivars to HS, respectively. CLN is reported to be tolerant to salt stress (Saeed *et al.*, 2011) and TMV infection (https://avrdc.org). CLN has been shown to have higher plant vigour and pollen viability as compared to sensitive CA4 (Sangu *et al.*, 2015). In addition, CLN is reported to maintain highest percentage of seeded fruits under HS as compared to several other cultivars (Comlekcioglu and Soylu, 2010). The CLN and CA4 pair has been used as donor parents for generating mapping populations towards HS (https://avrdc.org). Thus, decoding the transcriptome of these two cultivars in HS response should provide valuable resource for future research to improve thermotolerance in tomato.

### Distinct transcriptome signatures between two cultivars

Response to abiotic stress is a complex trait which involves a network of several genomic elements. Therefore, the transcriptome‐wide comparative landscape of the selected tolerant‐sensitive cultivar pair should shed insights into regulatory ‘hot spots’ to understand thermotolerance mechanism. Several studies have explored different time points and heat regimes to delineate the HS‐responsive genes (He *et al.*, 2019; Kang *et al.*, 2020; Larkindale and Vierling, 2008; Rao *et al.*, 2020; Wang *et al.*, 2018a, 2018b; Wang *et al.*, 2017). In nature, plants protect themselves from severe damage to extreme HS by acclimating themselves to sub‐lethal temperature stresses. To mimic the natural conditions in the study, the plants were first acclimated to HS by exposing them to milder HS followed by exposure to harsh temperatures (see Materials and Methods). More than 55 million good quality trimmed reads each for control and HS response were obtained, and the paired‐end data sets exhibited 95%–97% mapping on tomato genome in different data sets (Table S1). Out of 35 768 genic loci (ITAG release 3.2) of tomato, 26 631 genes were detected in the present study (Table S2). The remaining genes are either very low expressing or could be expressing in tissues other than leaves. PCA of transcriptome samples showed very good correlation between the biological repeat data sets and demonstrated a clear transcriptomic disparity between the two cultivars as well as distinctly separated the stress and control samples (Figure S2a). This was also substantiated by hierarchical clustering of the control and heat transcriptomic data sets; they being divided into two clusters (Figure S2b). The hierarchical clustering of 35 768 genes showed universal to cultivar‐specific HS regulation of several gene clusters (Figure S2c).

### Universal to cultivar‐specific regulation to HS

**Figure 2:**
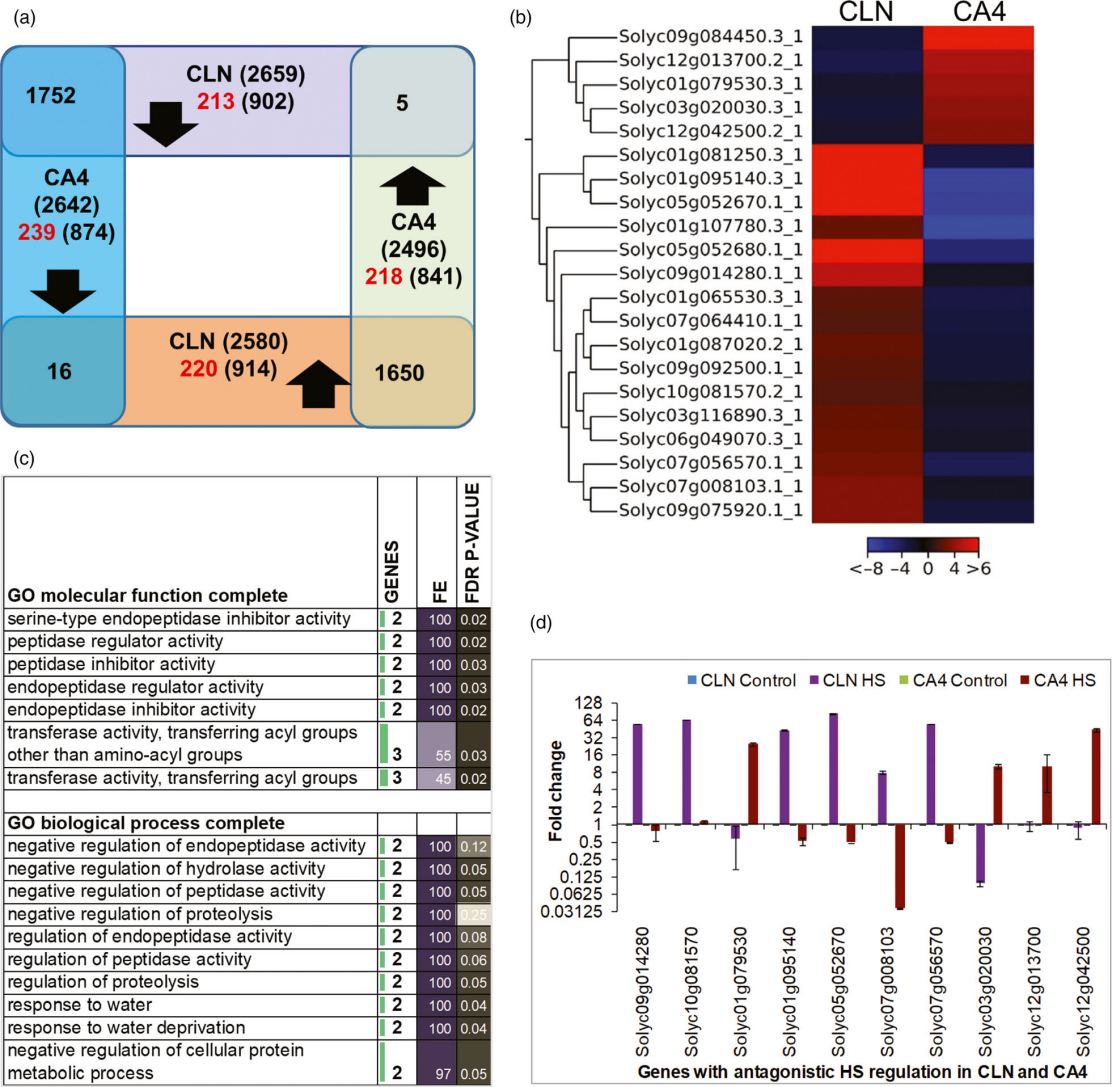
Heat stress‐responsive transcriptional modulations in leaf of tolerant, CLN and sensitive, CA4 tomato cultivars. (a) The Venn diagram depicts the comparison of heat response of CLN and CA4 leaf transcriptome. The genes with at least twofold up‐ or down‐regulation with FDR *P*‐value of ≤0.05 were considered. The genes with strict cultivar‐specific HS response are written in red. (b) The expression profile of cluster of genes with opposite heat‐mediated regulation in leaf of CLN and CA4 under control and heat conditions. Scale depicts the log2‐transformed fold change. (c) GO‐enrichment analysis of molecular function and biological process of the genes with inverse HS regulation in tolerant and sensitive cultivar following the PANTHER Overrepresentation Test. (d) The qRT‐PCR validation of selected genes with antagonistic HS response between CLN and CA4 in leaves. In qRT‐PCR, two biological replicates with three technical repeat and actin as endogenous control were used.

We next examined only that fraction of transcriptome that was significantly modulated (FDR *P*‐value ≤ 0.05 and fold change ≥2 and ≤−2 for up‐ and down‐regulated genes, respectively) in leaf under HS as depicted by volcano plots (Figure S2d). A total of 6954 differentially expressing genes (DEGs) were identified between both cultivars (Figure 2a). This number is the highest number of DEGs so far in tomato and the first for leaf utilizing contrasting cultivars (Bita *et al.*, 2011; Fragkostefanakis *et al.*, 2016; Fragkostefanakis *et al.*, 2015; Keller *et al.*, 2018). A slightly higher number of HS‐responsive genes were present in the HS‐tolerant cultivar (5239 vs. 5138 in CLN and CA4, respectively); moreover, the up‐regulated events were more in tolerant cultivar [2580 (CLN) and 2496 (CA4)], while almost equal number of down‐regulated genes were present in both cultivars [2659 (CLN) and 2642 (CA4)] (Figure 2a). A high magnitude of up‐regulation has also been reported in anthers of heat‐tolerant (Heat Set 1) tomato genotype in comparison with sensitive genotype, Falcorosso (Bita *et al.*, 2011). Surprisingly, only 21 genes showed an opposite (antagonistic) HS regulation between CLN and CA4 (Figure 2a,b). Five genes, viz. Solyc03g020030 (PIN‐type‐II proteinase inhibitor 69), Solyc09g084450 (proteinase inhibitor I), Solyc12g013700 (Stem‐specific protein tsjt1), Solyc01g079530 (RING/FYVE/PHD zinc finger superfamily protein) and Solyc12g042500 (gibberellin‐regulated family protein), were down‐regulated in CLN but were up‐regulated in CA4. There were 16 genes, viz. Solyc01g065530 (protein COBRA), Solyc01g081250 (glutathione s‐transferase), Solyc01g087020 (transmembrane protein), Solyc01g095140 (late embryogenesis abundant protein), Solyc01g107780 (glycosyltransferase), Solyc03g116890 (WRKY transcription factor 39), Soly05g052670, Solyc05g052680 and Solyc09g014280 (all three HXXXD‐type acyltransferase family proteins), Solyc06g049070 (nucleotide/sugar transporter family protein), Solyc07g008103 (blue copper protein), Solyc07g056570 (Notabilis), Solyc07g064410 (Kua‐ubiquitin conjugating enzyme hybrid), Solyc09g075920 (serine/threonine‐protein kinase), Solyc09g092500 (glycosyltransferase) and Solyc10g081570 (Marmande), were up‐regulated in CLN but down‐regulated in CA4 upon HS. Gene ontology (GO) enrichment of these antagonistic genes highlighted the significant enrichment of negative regulation of endopeptidase, peptidase and proteolysis, response to water, pollination and transferase activity (Figure 2c). Among these, five (the two proteinase inhibitors and the three HXXXD‐type acyltransferases) have roles in plant defence. The qRT‐PCR validation of 10 genes selected from the above clusters confirmed the opposite HS regulation in CA4 and CLN (Figure 2d).

Around ~49% (3402 out of 6954) HS‐responsive genes followed conserved HS‐regulated gene expression; 1752 DEGs were up‐regulated and 1650 DEGs were down‐regulated in both cultivars (Figure 2a), signifying a core universal HS response. GO enrichment analysis suggested the involvement of up‐regulated genes in cellular response to nutrient starvation specifically phosphate starvation in addition to protein folding (Figure S3a,b). Furthermore, proteins belonging to chaperones, winged helix TFs, mRNA splicing and processing factors were significantly enriched among the conserved up‐regulated genes (Figure S3c). KEGG pathways like protein processing in ER, spliceosome and ubiquitin‐mediated proteolysis were enriched in conserved up‐regulated genes (Table S3). Many recent reports on stress‐responsive regulation of spliceosome per se, as well as, crucial role of alternatively spliced transcripts in response to abiotic stresses including HS have been reported in plants (Deng *et al.*, 2011; Seo *et al.*, 2012, 2013; Carrasco‐López *et al.*, 2017; Calixto *et al.*, 2018; Filichkin *et al.*, 2018; Laloum *et al.*, 2018; Huertas *et al.*, 2019). The unfolded protein response (UPR) is a conserved response that is elicited by ER stress and is known to protect plants from adverse environmental stresses (Park and Park, 2019; Wan and Jiang, 2016). On the other hand, wide range of biological processes and metabolic pathways belonging to various protein classes were down‐regulated in both the cultivars (Figures S4–S6; Table S3). We found that 26 genes were highly up‐regulated (≥100‐fold) in both cultivars but none followed similar folds down‐regulation in both (Table S2). The highly expressed genes (>1000‐fold) in CLN and CA4 belonged to the HSP protein family (Solyc03g113930.2.1, Solyc01g102960.3.1, Solyc03g082420.3.1, Solyc11g020330.1.1) and a serine carboxypeptidase (Solyc04g077640.3.1).

Nearly 51% (3531) of HS‐responsive genes followed cultivar‐biased stress regulation, that is, those DEGs which were significantly up‐ or down‐regulated in only one cultivar but not in the other cultivar. These included 1816 (914 up‐regulated and 902 down‐regulated) DEGs in CLN and 1715 (841 up‐regulated and 874 down‐regulated) DEGs in CA4 (Figure 2a). To further discriminate the true cultivar‐biased HS‐responsive DEGs, the above genes were filtered out by adopting strict parameters (See Materials and Methods). As a result, 218 up‐ and 239 down‐regulated genes showed CA4‐specific HS regulation, while 220 up‐ and 213 down‐regulated genes followed CLN‐specific HS response (Figure 2a; Table S2). Genes exhibiting the CLN‐specific down‐regulation were enriched in terms associated with ‘photosynthesis’ in biological process and cellular component categories, while glycopeptide alpha‐N‐acetylgalactosaminidase activity, cysteine synthase activity, chlorophyll binding, and ATPase activity were enriched among the molecular function category (Figure S7). For genes exhibiting the CLN‐specific up‐regulation, there was no significant enrichment, while there was significant enrichment of translation, ribosomal proteins, ribosome, structural constituents of ribosomes, etc., in CA4‐specific up‐regulated genes (Figure S8). Role of calcium‐mediated signalling and GTPase activity was highlighted among the CA4‐specific down‐regulated genes (Figure S9).

In addition, some metabolic pathways were enriched specifically in up‐regulated genes in CLN only, *viz.* MAPK signalling pathway, circadian rhythm, plant hormone signal transduction, sulphur metabolism and glycerolipid metabolism (Table S3). Tomato, MPK1 regulates thermotolerance via SlSPRH1 involved in antioxidant defence (Ding *et al.*, 2018). Phosphorylation is a critical regulatory mechanism for several HSFs (Evrard *et al.*, 2013; Link *et al.*, 2002). The circadian clock genes sustain healthy and accurate timing over an array of physiological temperatures in different plant species. HSFB2b‐mediated transcriptional repression of PRR7 is known to direct HS responses of the circadian clock in *Arabidopsis* (Kolmos *et al.*, 2014). Furthermore, ZEITLUPE and HSP90 are critical for the maintenance of thermo‐responsive protein quality (Gil *et al.*, 2017; Gil and Park, 2017). Our data contribute to identification of HS‐responsive signature genes which can be exploited for thermotolerance augmentation in tomato and other crop plants. A comparative transcript profiling in more time points and stress regimes could lead to further insights into deciphering core thermotolerance mechanism.

### Distinct modulation of transcription factors in CLN and CA4 under heat stress

**Figure 3:**
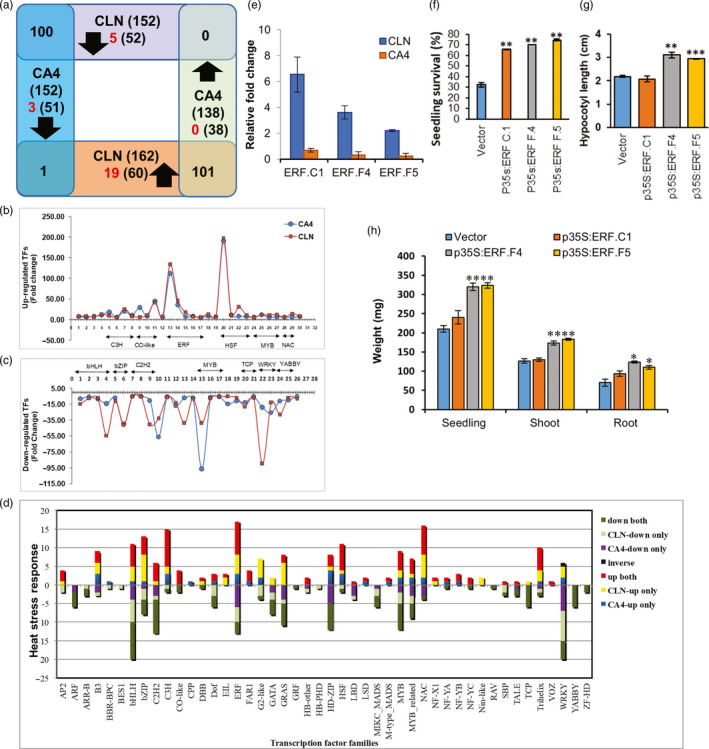
Heat stress‐mediated regulation of transcription factors in leaf of contrasting cultivars. (a) Venn diagram showing the common to specific heat responsiveness of transcription factors in leaf of tolerant, CLN and sensitive CA4 cultivar. (b,c) The plots showing the highly up‐regulated [(b), FC ≥ 5; FDR *P*‐value ≤ 0.05] and down‐regulated [(c), FC ≤ −5; FDR *P*‐value ≤ 0.05] transcription factors in CLN and CA4 in response to heat stress. (d) The comparison of cultivar‐specific heat response of various transcription factor families in leaf of tomato. (e) Expression analysis of ERFs: ERF.C1, ERF.F4 and ERF.F5 in response to HS in leaf of CLN (tolerant) and CA4 (sensitive) cultivar by qRT‐PCR. (f,g) Percentage seedling survival after HS for vector control and different p35S:ERFs overexpressing seedlings and comparative morphological analysis using hypocotyl length (g), seedling weight, shoot weight and root weight (h). The data were calculated 6 days postheat stress imposition from four biological sets of 70 seedlings for each gene. Error bars denote standard error. *P < 0.05, **P < 0.01 and ***P < 0.001.

Gene regulation by TFs acts as focal nodes in all molecular networks. We predicted 1963 tomato TFs belonging to 58 families, using the TF prediction server of PlantTFDB (http://planttfdb.cbi.pku.edu.cn/prediction.php) with protein sequences of 35768 genes (ITAG version 3.2) as query (Table S4). Out of these, 402 TFs belonging to 46 families were found to be heat‐responsive in at least one cultivar. In CLN and CA4, 314 (152 down/162 up) and 285 (152 down/133 up) TFs were significantly differentially regulated, respectively (Figure 3a, Table S4). Similar HS response was evident for 201 (100 down/101 up) TFs in both cultivars (Figure 3a). Thirty‐seven TFs (29 and 16 in CLN and CA4, respectively) exhibited very strong heat induction (FC > 10). Thirty TFs followed conserved high (FC > 5) up‐regulation in both the cultivars, while 26 followed high (FC < −5) down‐regulation in both the cultivars. These highly transcribed TFs include members belonging to ERF (5 members), CO‐like (3 members), HSFs (4 TFs); 4 members each of MYB and C3H, C2C2‐CO‐like and NAC (2); and 1 each of B3, bHLH, bZIP, C2H2, Dof1, FAR1, HD‐ZIP1 and NF‐YB D (Figure 3b). Of these, 3 TFs, viz. HSFA2 (Solyc08g062960), and dehydration‐responsive element‐binding (DREB) TF (Solyc05g052410) were more than 100‐fold up‐regulated in both cultivars. The plot of above 30 TFs clearly revealed that the heat‐tolerant CLN showed higher magnitude of HS‐mediated induction in majority of the TFs and common key TF pool was activated upon HS in both cultivars (Figure 3b). Moreover, 26 TFs showed HS‐mediated strong repression (FC ≤ 5) in both cultivars (Figure 3c). While Solyc08g008280 (WRKY‐53) was the top repressed candidate in CLN, Solyc10g005080 (late elongated hypocotyl) was highly down‐regulated TF in CA4 under HS (Figure 3c). In addition, other TFs exhibiting strong repression belong to TF families bHLH (4), bZIP (2), C2H2 (3), MYB (3), TCP (2), WRKY (2) and YABBY (2) (Figure 3c and Table S4). The highly HS‐responsive TF families with more than 20 of its members significantly modulated (up‐regulated and down‐regulated) at transcript level include bHLH, bZIP, C2H2, C3H, ERF, GRAS, HD‐ZIP, HSF, MYB, NAC and WRKY (Figure 3d). Further, we scanned for those TF families that had at least two differentially regulated members in response to HS and also had ≥80% of its members exhibiting either up‐ or down‐regulation. Using these criteria, members belonging to TF families B3, C3H, HSF, NAC and Trihelix were majorly induced in response to HS, while ARF, Dof, MADS, TCP and YABBY were primarily repressed in response to HS (Figure 3d). Thermotolerance in transgenic systems has been achieved by modulating the levels of several TFs like ZAT12 (Shah *et al.*, 2013), NAC019 (Guan *et al.*, 2014), bZIP28 (Srivastava *et al.*, 2014) and PIF4 (a bHLH transcription factor) in Arabidopsis (Gangappa *et al.*, 2017; Hwang *et al.*, 2017).

The most studied and well‐established TF family in response to HS is the HSF family. Seven tomato HSFs (A1b, A2, A3, A4c, A5, A7 and C1) exhibited HS‐mediated up‐regulation in both the cultivars with highest up‐regulation for HSFA2 (Table S4). We noted that the repressor class B HSF members, namely HSFB1, B2a and B2b, are up‐regulated only in the sensitive CA4. Whether this has any direct correlation with CA4 being HS sensitive needs to be further evaluated. HSFB1 is endowed with both co‐activator as well as repressor functions in tomato, while it acts as repressor in Arabidopsis (Bharti *et al.*, 2004; Fragkostefanakis *et al.*, 2019; Ikeda *et al.*, 2011). The Arabidopsis *hsfb1‐hsfb2b* double knockout mutant demonstrates higher acquired thermotolerance (Ikeda *et al.*, 2011; Kumar *et al.*, 2009). HSFA6a was up‐regulated, and HSFA4b was down‐regulated under HS only in CLN but not in CA4 (Table S4). AtHSFA6a is induced in response to ABA, salt and drought and is responsible for the transcriptional activation of stress‐responsive gene DREB2a via ABA‐dependent signalling pathway (Hwang *et al.*, 2014, 2017). Furthermore, we investigated the ERF family that showed highest number of HS‐responsive TFs and include AP2, DREBs and ERFs (Sakuma *et al.*, 2002). Various ERFs are reported to mediate development and stress response, but only few address their regulatory role in thermotolerance (Licausi *et al.*, 2013; Klay *et al.*, 2014, 2018; Müller and Munné‐Bosch, 2015). Out of 147 ERFs reported in tomato, 30 members followed differential HS response in CLN and CA4 (Table S4). Of these, 12 ERFs were similarly regulated (9 up‐regulated and 3‐regulated down) in both cultivars, while others were regulated in cultivar‐biased manner. We selected three previously uncharacterized ERFs (ERF.F4, ERF.F5, and ERF.C1) in HS response based on the observation that they all maintained high levels in CLN and low levels in CA4 under HS. This was validated by qRT‐PCR also (Figure 3e). These 3 ERFs are reported in fruit development (Hao *et al.*, 2015; Di Matteo *et al.*, 2013) in tomato, but their role in thermotolerance was lacking. Functional characterization of the above three ERFs by transient overexpression significantly enhanced (more than doubled) the tomato seedling survival rate under HS as compared to vector control plants (Figure 3f) with highest survival percentage for ERF.F5. Seedlings overexpressing ERF.F4 and F5 exhibited higher hypocotyl length as well as shoot, root and total seedling weight as compared to vector control plants (Figure 3g,h). Our data on TF regulation dynamics highlight role of TF families other than the undisputed relevance of HSFs in HS response. These TFs could be promising candidates for enhancing tomato thermotolerance as we demonstrated for the three ERFs that function as positive regulators of HS response.

### In‐planta characterization of novel regulators for thermotolerance

Cultivar‐specific gene regulation is critical to delineate regulatory networks involved in tolerance mechanisms under varied environmental conditions (Balyan *et al.*, 2017; Boccacci *et al.*, 2017; Koeslin‐Findeklee *et al.*, 2014; Sun *et al.*, 2010). This study identified 21 antagonistically expressing genes upon HS in the two contrasting varieties, the differential expression of 10 of which was also corroborated by qRT‐PCR (Figure 2d). Surprisingly, the direct role of these genes in hyperthermal stress tolerance is either totally lacking or very limited. Therefore, to establish the regulatory influence of these genes in governing thermotolerance, we performed *in‐planta* functional analysis using overexpression and virus‐induced gene silencing (VIGS) in tomato. To shortlist few candidate genes for this analyses, we put another filter by assessing the transcript abundance of the above 10 genes under HS in another pair of contrasting cultivars, namely Pusa Sadabahar from the tolerant clade and Pusa Ruby from the sensitive clade (Figure S10 and Figure 1). We selected two genes (Solyc09g014280 and Solyc07g056570) that are up‐regulated in CLN as well as Pusa Sadabahar while are down‐regulated in both sensitive cultivars (‘tolerant‐up genes’) as well as one gene (Solyc03g020030) which is highly down‐regulated in tolerant CLN but up‐regulated in both sensitive cultivars (‘sensitive‐up gene’).

### Knocking down ASATs decreases thermotolerance

**Figure 4:**
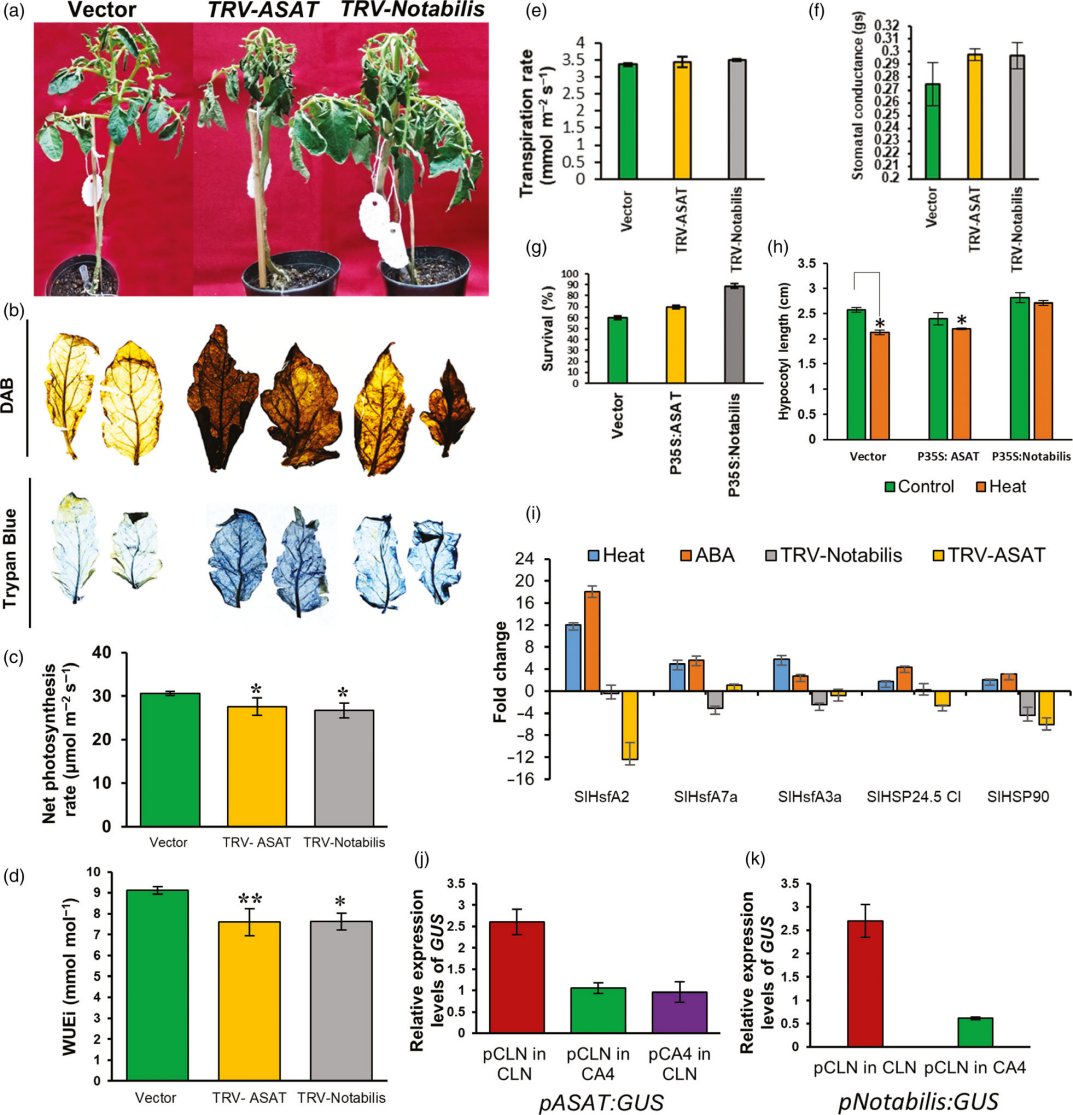
Functional validation of the roles of *ASAT* and *Notabilis* genes in response to heat stress. Functional validation of the roles of *ASAT* and *Notabilis* genes in response to heat stress. (a) Phenotypes of HS‐treated empty vector (TRV), TRV‐ASAT and TRV‐Notabilis‐silenced plants. 15‐day‐old CLN plants were agro‐infiltrated with empty vector (TRV), TRV‐ASAT and TRV‐Notabilis VIGS constructs. Plants were given heat stress 3 weeks postinfiltration. Survival was gauged 6 days postrecovery. (b) DAB and Trypan blue staining of leaves of HS‐treated empty vector (TRV), TRV‐ASAT and TRV‐Notabilis‐silenced plants. (c–f) Estimation of net photosynthesis rate (μmol/m^2^/s), water‐use efficiency (mmol/mol), transpiration rate (mmol/m^2^/s) and stomatal conductance (mol/m^2^/s) in empty vector (TRV), TRV‐ASAT and TRV‐Notabilis‐silenced plants following heat stress. (g,h) Estimation of survival (percentage) and percentage reduction in hypocotyl length of seedlings under control conditions and after 5 days of heat stress in plants with overexpression of empty vector, ASAT and Notabilis seedlings. Data are means of four biological sets of 70 seedlings each. (i) Expression profiles of HSR genes in heat, ABA and VIGS silenced ASAT and Notabilis plants by qRT‐PCR. (j) qRT‐PCR‐based GUS:reporter assays of CLN and CA4 ASAT promoters in CLN and CA4 background. (k) qRT‐PCR‐based GUS:reporter assays of CLN:Notabilis promoter in CLN and CA4 background. Error bars denote standard error. *P < 0.05, **P < 0.01 and ***P < 0.001.

*Solyc09g014280* gene codes for an *HXXXD‐type acyltransferase* and belongs to the acylsugar acyltransferases (ASATs) class of the large and diverse BAHD family of acyltransferases (Moghe *et al.*, 2017). Acylsugars are a group of small specialized metabolites having diverse structures that are restricted to plants in the Solanaceae family that act as natural chemicals against pests like whiteflies and spider mites (Alba *et al.*, 2009; Liedl *et al.*, 1995). Acylsugars are produced in the tip cell of the long glandular secreting trichomes using ASAT enzymes that catalyse sequential addition of specific acyl chains to the sucrose molecule using acyl CoA donors (Maldonado *et al.*, 2006; Schilmiller *et al.*, 2008). *Solyc09g014280 and Solyc05g052670* (another tolerant‐up gene but not being functionally characterized; Figure S10) are reported to be enriched in the glandular trichomes in tomato (Moghe *et al.*, 2017). While *Solyc09g014280* is induced upon potato cyst nematode infection in tomato roots (Swiecicka *et al.*, 2017), *Solyc05g052670* is up‐regulated in tomato in response to tomato yellow leaf curl virus infection (Chen *et al.*, 2013). Here, we report yet unexplored function of an ASAT member in tomato HS response. Plants exhibiting successful knock‐down of *Solyc09g014280* transcripts (Figure S11) showed severe drooping of leaves upon recovery after being exposed to HS in comparison with vector control plants (Figure 4a). Heat stress results in a higher than optimal concentration of ROS that not only affect plant’s ability to photosynthesize but also cause cell death. These parameters are routinely adopted to assess thermotolerance of plants*.* The suppression of *ASAT* gene shows enhanced hydrogen peroxide (one of several reactive oxygen species) levels as well as cell death under HS as judged by DAB staining and Trypan blue staining, respectively (Figure 4b). Gas exchange parameters measured by LI‐COR 6400 portable photosynthesis measuring system at the end of the heat treatment of *ASAT‐*silenced plants showed significant drop in net photosynthesis rate and water‐use efficiency (WUEi) as well as enhanced stomatal conductance and transpiration rate (Figure 4c–f) highlighting the role of ASAT as a positive regulator of thermotolerance. This conclusion was further strengthened by assessing seedling performance under HS when *ASAT* gene was transiently overexpressed. The seedling survival increased by nearly 17% in comparison with vector control (Figure 4g), and the reduction in hypocotyl length under HS was also around 8% in overexpression plants in comparison with 18% in vector control plants (Figure 4h). Moreover, the relative abundance of HS marker genes (HSFA2a/A7a/A3a, sHSP24.5 CI and HSP90) (Figure 4i) was reduced several folds in *ASAT*‐silenced plants reiterating the fact that ASAT has a key role in providing thermotolerance. Further, to understand how the transcriptional regulation of *ASAT* results in antagonistic expression between the contrasting cultivars, we sequenced 1.5 kb promoter from both the cultivars and checked promoter ASAT:GUS–reporter expression by assaying CLN promoter:GUS–reporter in CA4 background and vice versa. We found that there was one substitution of ‘T’ to ‘A’ in CA4 promoter, which disrupted the binding sites of two TFs, namely NF‐Y A/B/C and AP2 having *cis*‐binding sites, that is ACAAT and AATCAA, respectively, in CA4. If this mutated regulatory site abrogates TF binding and in turn *ASAT* expression in CA4 then the expression of CA4‐ASAT promoter:GUS in CLN background is expected to reduce, indeed this was the case (Figure 4j). We found that the expression of CLN‐ASAT promoter:GUS was up‐regulated in CLN background (Figure 4j, Figure S12), but not in CA4 background. It appears that in addition to the promoter sequence context, cultivar‐specific differential TF pool between CLN and CA4 also regulates the expression of this gene. *ASAT* promoter has binding sites for more than 15 different TFs (Figure S13). Many of these TFs showed differential expression pattern between the two cultivars (Table S4 and Figure 3d). Our data demonstrated a novel role of ASAT as a positive regulator of tomato thermotolerance. The exact mode of action of this enzyme under HS needs further attention.

### Silencing Notabilis confers thermo‐sensitivity in tomato

We then functionally validated the other tolerant‐up gene, *Solyc07g056570* or *Notabilis* (9*‐cis‐epoxycarotenoid dioxygenase 1*). Notabilis regulates the rate‐limiting step in the ABA biosynthesis pathway and has important role in water stress tolerance as evidenced by several reports (Burbidge *et al.*, 1999; He *et al.*, 2018; Luchi *et al.*, 2001; Qin and Zeevaart, 1999; Wan and Li, 2006). In Arabidopsis, ABA‐deficient and signalling mutants are involved in acquired thermotolerance acquisition (Larkindale and Huang, 2004). *Arabidopsis thaliana* NGATHA1 induces ABA biosynthesis by activating *Notabilis* homolog *NCED3* gene during dehydration stress (Sato *et al.*, 2018). In lettuce, its homolog (*NCED4*) is the causal gene in the Htg6.1 QTL, associated with thermo‐inhibition of seed germination (Huo *et al.*, 2013). Here, we investigate the role of Notabilis in tomato thermotolerance. When *Notabilis* knocked‐down plants (Figure S11) were exposed to HS and assessed after recovery, they exhibited decreased thermotolerance as evidenced by the morphological (severe wilting of leaves) phenotype (Figure 4a). This is in accordance with the phenotype observed in the tomato null mutant of *notabilis* that is deficient in ABA synthesis (Burbidge *et al.*, 1999; Thompson *et al.*, 2000, 2004). Reduced thermotolerance was also reported in Arabidopsis mutants with impaired *NCED6* and *NCED9* expression (Gabriele *et al.*, 2010). It is known that high ABA levels cause ROS outburst. However, the leaves of *Notabilis‐*silenced plants show slightly increased ROS levels and HS‐induced cell death in comparison with vector control plants (Figure 4b). This apparent anomaly may be attributed to a balance between low ROS levels due to reduced ABA levels and other pathways contributing to ROS production in response to HS. The gas exchange analyses further supported the important role of *Notabilis* in thermotolerance. There was a significant reduction in the photosynthetic rate and water‐use efficiency along with a significant rise in stomatal conductance and enhanced transpiration rate in silenced plants in contrast to vector control plants (Figure 4c–f). Overexpression of wheat *TaNCED* gene in Arabidopsis enhanced tolerance to drought stress by reducing stomatal conductance and transpiration (Tong *et al.*, 2017). Further proof for *Notabilis* as a gene imparting thermotolerance was provided from tomato plants overexpressing *Notabilis* as judged by better seedling survival percentage and hypocotyl length in comparison with vector control (Figure 4g,h). We then measured the relative abundance of HS marker genes in the silenced *Notabilis* plants; the level of all the genes was reduced several folds upon HS in comparison with control (Figure 4i). Moreover, when we checked the expression of these HSR genes in response to ABA, they were all up‐regulated, suggesting *Notabilis* as an upstream regulator of these HS marker genes (Figure 4i). Of these HSFs, HSFA2a and HSFA7a were down‐regulated the most and appeared as the critical candidates in the Notabilis‐ABA pathway‐mediated thermotolerance. Furthermore, we asked whether the opposite expression of *Notabilis* in CLN‐CA4 cultivars is because of variation in promoter sequences and/or an inherent difference in TF pool of both the cultivars. Sequence information of promoters for both the varieties highlighted no SNPs; thus, a regulatory influence of the *cis*‐sites in the contrasting expression was ruled out. We then checked the expression of GUS gene hooked to *Notabilis* promoter in CLN and CA4 background. Figure 4k and Figure S12 highlight that GUS expression is up‐regulated only in CLN background, pointing to a probable cultivar‐specific TF‐mediated regulation. In the literature, *Notabilis* is shown to be transcriptionally regulated by SlNAP2 (Solyc04g005610) (Ma *et al.*, 2018) a NAC TF. Indeed, this TF is up‐regulated in CLN (~2‐fold) but not in CA4 in response to HS (Table S2).

### Silencing PI‐II gene enhances tomato thermotolerance

**Figure 5:**
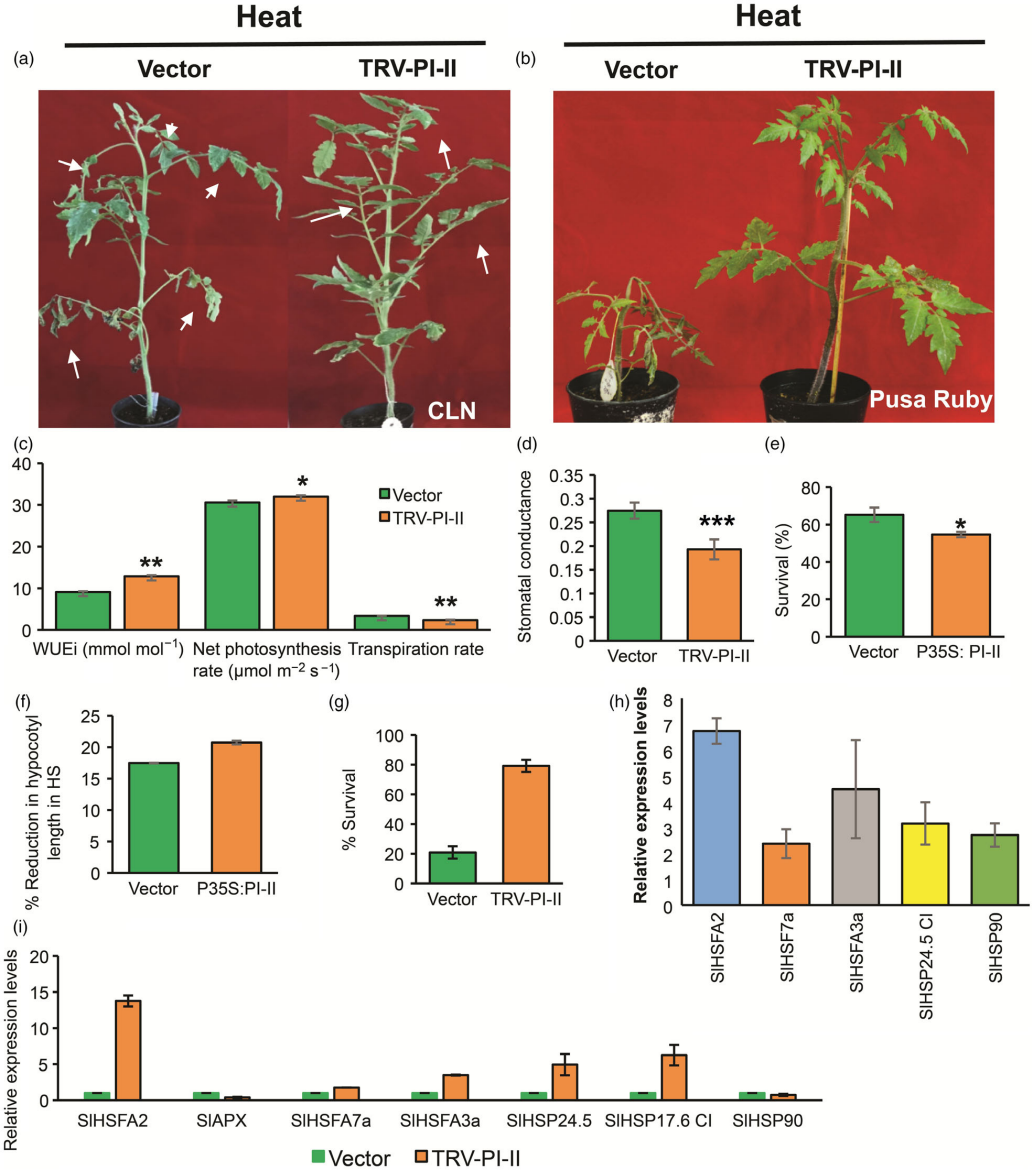
Functional validation of Pin‐II type proteinase inhibitor in response to HS. (a,b) 15‐day‐old tomato plants were agro‐infiltrated with empty vector (TRV) and TRV‐pin‐II type proteinase inhibitor VIGS construct (TRV‐PI‐II). Plants were given heat stress 3 weeks post‐agro‐infiltration. Survival was gauged 6 days postrecovery. Phenotypes of HS‐treated TRV and TRV‐PI‐II plants in the tolerant cultivar, CLN (a) and in the sensitive cultivar, Pusa Ruby (b). White arrows in (a) show phenotypic severity postheat stress. (c) Estimation of water‐use efficiency (mmol/mol), net photosynthesis rate (μmol/m^2^/s), transpiration rate (mmol/m^2^/s) and (d) stomatal conductance (mol/m^2^/s) in TRV and TRV‐pin‐II type proteinase inhibitor plants following heat stress of CLN plants. (e) Estimation of seedling survival (percentage) and (f) percentage reduction in hypocotyl length after HS in vector control and Pin‐II type proteinase inhibitor overexpression seedlings. (g) Estimation of survival rate in empty vector (TRV) and TRV‐pin‐II type proteinase inhibitor VIGS silenced plants in Pusa Ruby after heat stress. (h,i) Expression profiles of HSR genes in VIGS silenced PI‐II plants in tolerant cultivar CLN (h) and in sensitive cultivar Pusa Ruby (i). Data are means and SE of four biological sets of 70 seedlings each. *P < 0.05, **P < 0.01 and ***P < 0.001.

Furthermore, another gene but a ‘sensitive‐up gene’ which is *Solyc03g020030* was functionally characterized. This belongs to the proteinase inhibitor‐II (PI‐II) serine‐PI family having dimeric domains in the protein, inhibiting trypsin and chymotrypsin (Tamhane *et al.*, 2012). The PI‐IIs are well documented in plant defence against insects, bacteria and fungi (Rehman *et al.*, 2017). Few reports in abiotic stress tolerance like drought, salinity, osmotic variations and pH are also documented (Huang *et al.*, 2007; Shan *et al.*, 2008; Srinivasan *et al.*, 2009). Only a Kunitz PI (another class of serine‐PI family) has been shown to be induced in response to heat stress (Annamalai and Yanagihara, 1999), however, information regarding PI‐II‐class role in HS is completely lacking. Solyc03g020030 was expressed at high levels in CLN under control condition but decreased up to threefold under HS, while significant increase was noticed in CA4 under HS. We find there is enrichment of GO term of ‘serine‐type endopeptidase inhibitor activity’ in CA4‐specific up‐regulation (Figure 2c). Silencing of *PI‐II* was confirmed by qRT‐PCR (Figure S11), and the leaves of knock‐down plants were upright, enduring HS better than those of vector control plants which showed drooping and burnt leaves (Figure 5a). Moreover, there was appreciable difference phenotypically when VIGS was repeated in sensitive background (Pusa Ruby) (Figure 5b). The CLN *PI‐II* knock‐down plants exhibited significant enhancement in WUEi and net photosynthetic rates as compared to TRV‐control plants under HS (Figure 5c). In addition, both the transpiration rate and the stomatal conductance were lower in TRV‐PI‐II plants under HS (Figure 5c,d). The *PI‐II* overexpression in CLN decreased the tolerance of the seedlings of this robust cultivar as evident from reduced hypocotyl length as well as survival rate under HS in comparison with vector control seedlings. In contrast, VIGS‐based silencing of *PI‐II* in Pusa Ruby exhibited higher survival rate (Figure 5e–g). Furthermore, enhanced expression of HS marker genes in *PI‐II‐*silenced plants confirmed its role as a negative regulator of HS tolerance (Figure 5h,i). In tomato, SlbZIP1 TF (Solyc01g079480.3) transcriptionally regulates *PI‐II* (Zhu *et al.*, 2018). When we checked our NGS data for a possible cultivar‐specific differential response of *PI‐II* by SlbZIP1 in response to HS, the TF was not significantly differentially regulated in CLN (1.85‐fold change) and CA4 (1.25‐fold change) (Table S2). This suggested that HS‐mediated regulation of *PI‐II* is governed by other TFs or may have epigenetic regulation. The down‐regulation of *PI‐II* by VIGS established its role in retrograde signalling in the regulation of HS. Knocking down this gene by CRISPR/RNAi technology could be highly promising for engineering for HS‐thermotolerance.

## Experimental procedures

### Screening of tomato cultivars for HS tolerance

Nine contrasting tomato cultivars (Table S5a) including five HS‐tolerant [CLN1621L, IIHR2201, IIHR2274, Pusa Sadabahar and Hisar Arun] and four sensitive cultivars (CA4, Pusa Ruby, Pusa Rohini and Pusa120) were screened under natural HS as well as controlled HS conditions. The cultivars were screened under natural warming conditions to assess the effect of HS on the fruit set and yield‐related traits. Thirty to forty seedlings of each cultivar were grown in field under optimal growing season (4th week of October 2014/2015; mean day/night temperature: 23.4/17.5 °C) and warmer climatic conditions (4th week of February 2015/2016; mean day/night temperature: 35.5/30.3 °C) (Figure S14). The average maximum temperature in field during the flowering (April) and fruit set periods (May) was 34.5 °C and 39 °C, respectively. Randomly selected 20 plants were evaluated for each cultivar under control and HS condition. Data were collected after red‐ripe fruit stage for the assessment of variation in yield (average of red‐ripe fruit weight/plant) and fruit set (average of total number of flowers converted to fruits/plant × 100) among the genotypes.

The screening was also performed under controlled HS conditions at seedling (5‐day‐old) and 1‐month‐old plants by survival assays and detailed physiological analysis. For the survival assays, seeds were germinated on filter paper soaked with deionized water at 26 °C. Four technical replicates (30 seedlings each) of each cultivar were subjected to basal HS at 45 °C for 4.5 h. Survival (percentage) was evaluated after overnight recovery. The experiments were repeated three times independently. For survival assay in 1‐month‐old plant, 5‐day‐old seedlings with uniform growth were transplanted in plastic pots, filled with soilrite and placed in a plant growth chamber (26/21 °C; day/night: 16/8 h; relative humidity: 60%; light intensity: 300 µm/m^2^/s). One‐month‐old plants were gradually acclimated to heat stress (26 to 45 °C in a time span of 4 h), followed by 4.5‐h incubation at 45 °C. Leaves from these experimental plants were harvested immediately after stress for measuring physiological parameters. For survival assay, five plants of each cultivar were evaluated for survival percentage after overnight recovery. The experiment was repeated two times independently. Relative water content (RWC), electrolyte leakage (EL) and proline content were determined as described by Houimli *et al. *(2010) and Bates *et al. *(1973). These experiments were repeated three times with five technical replicates each. Based on the performance of each cultivar, the percentage values of each trait were calculated under HS relative to respective controls which were set as 100%. All these traits were analysed by clustering and principal component analysis (PCA) using ClustVis online tool (Metsalu and Vilo, 2015). Clustering was performed using Manhattan distance and complete linkage. Unit variance scaling was applied to rows, and singular value decomposition (SVD) with imputation was used to calculate principal components.

### Transcriptome generation and analysis

The 1‐month‐old CLN (tolerant) and CA4 (sensitive) plants were acclimated to gradual increase in temperature from 26 °C to 45 °C for 4 h and then exposed to 45 °C for 4.5 h, followed by leaf tissue collection. High‐quality RNA isolated from leaf was used to construct paired‐end RNA‐Seq libraries and sequenced on Illumina platform with three biological replicates each from control and HS conditions for CLN and CA4. High‐quality 75 bp paired‐end raw reads were analysed by RNA‐Seq plug‐in of CLC genomics workbench version 9 using the tomato cDNA sequences from ITAG version 3.2 (https://solgenomics.net/organism/Solanum_lycopersicum/genome) as the reference on default parameters [mismatch cost: 2, insertion cost: 3, deletion cost: 3, length fraction: 0.8, global alignment: no, auto detect pair distance: yes, strand specificity: both, maximum number of hits for a read: 10, expression value: RPKM (Reads Per Kilobase of transcript per Million mapped reads)]. The expression data were analysed by the ‘Empirical analysis of Differentially Expressed Genes (DEGs)’ algorithm of CLC genomics workbench to perform the multi‐group comparison following exact test (Robinson and Smyth, 2008) in the EdgeR bioconductor package using default settings [total count filter cut‐off for common dispersion: 0.1, tag wise dispersion estimation: exact test comparison: all pairs, false discovery rate (FDR)‐corrected *P*‐values]. Volcano‐plot analysis was done by following the Gaussian statistical analysis on log_2_‐transformed RPKM values with FDR‐corrected *P*‐values (inhomogeneous *t*‐test). The hierarchical clustering of features/sample was performed following the Euclidean distance algorithm with average linkage. Principal component analysis (PCA) of all the RNA‐Seq data sets was performed using CLC genomics workbench version 9. The genes with dispersion fold change of ≥2 or ≤−2 with FDR correction *P*‐value of ≤0.05 (control vs. HS) were considered as heat responsive. The GO‐enrichment analysis was performed using the ‘Gene list analysis’ tool of PANTHER classification system (www.pantherdb.org, version 14.1) following the statistical over‐representation test using default settings (Bonferroni correction *P*‐value ≤0.05; fold enrichment ≥2). The KEGG pathway enrichment analysis was done using ShinyGO version 0.60 (*P*‐value ≤ 0.05). The 1kb upstream regulatory region of genes was retrieved from Sol Genomics Network (https://solgenomics.net) and analysed for cis‐regulatory elements using the Plant Promoter Analysis Navigator (PlantPAN; http://plantpan2.itps.ncku.edu.tw/) using multiple promoter analysis tool. The transcription factors were predicted using the TF prediction tool of PlantTFDB (http://planttfdb.cbi.pku.edu.cn/prediction.php) with protein sequences (ITAG version 3.2) as query.

### Quantitative real‐time PCR (qRT‐PCR)

RNA isolation, cDNA synthesis and qRT‐PCR were performed as per Paul *et al. *(2016). The expression assays were performed with at least two biological and three technical replicates and normalized to tomato actin gene as endogenous control (Han *et al.*, 2020; Qiu *et al.*, 2016; Yan *et al*, 2020) using comparative ∆∆CT method.

### Virus‐induced gene silencing (VIGS)

The silencing *via* VIGS was performed as described by Senthil‐Kumar and Mysore (2014). The partial coding region (300–400) of Solyc09g014280, Solyc07g056570 and Solyc03g020030 was designed using SGN VIGS tool (https://vigs.solgenomics.net/), amplified and cloned into pTRV2 vector followed by confirmation by sequencing. The positive clones were transformed into *Agrobacterium tumefaciens* GV3101 strain. About 15‐day‐old tomato plants were inoculated with a mixture of *A. tumefaciens* strains containing the pTRV2 vector constructs and pTRV1 into the first true leaf and the second true leaf as described previously (Senthil‐Kumar and Mysore, 2014). Empty pTRV2 vector was used as a control. Infected plants were incubated in the dark and moist conditions for 3 days and then transferred to the light. HS was imposed after 3 weeks at 45 °C for 4.5 h. Leaves were collected and frozen for qRT‐PCR analysis. Survival percentage was assayed after six days of recovery. The experiment was performed in three biological replicates with four plants per replicate for each gene.

### Transient overexpression and thermotolerance assay for tomato seedlings

The transient overexpression of selected genes followed by thermotolerance assays was standardized for different tomato cultivars by following the methodology adopted by Queitsch *et al. *(2000) with modifications. The CDS region of selected genes was cloned in pBI121 and transformed in *A*. *tumefaciens* EHA105 strain. Tomato seeds were allowed to germinate on wet paper towels in Petri dishes in the dark at 26 °C. Four‐day‐old seedlings were vacuum infiltrated with positive *A. tumefaciens* strain EHA105, containing the empty pBI121 vector or pBI121‐gene constructs. Before infiltration, EHA105 culture was grown up to OD_600_ = 1 at 28 °C by shaking (200 rpm) on incubator followed by centrifugation at 4500 rpm for 10 min. Pellet was further re‐suspended in infiltration buffer (1 m MgCl_2_: 100 µL, 1 m MES pH 5.7: 2.5 mL, D‐glucose: 250 mg, 100 mm acetosyringone: 2.5 µL) and kept for induction for 3–6 h at 23–26 °C. The final OD_600_ = 1 was obtained in desired volume with infiltration buffer. Seedlings were infiltrated at 500 mm Hg pressure for 15 min using a vacuum pump. Transiently transformed seedlings were placed at 26 °C for 2 days under controlled conditions followed by HS at 45 °C for 4.5 h. Plants were allowed to recover for 6 days at 26 °C followed by estimation of survival rate and hypocotyl length. Experiment was repeated four times with similar parameters with sample size of 70 seedlings per replicate.

### Gas exchange measurements

Tomato leaf gas exchange measurements, including water use efficiency (WUEi), net photosynthetic rate (A), transpiration rate (E) and stomatal conductance (Gs), were measured simultaneously by using a portable LI‐COR 6400 photosynthesis system (LI‐6400; Li‐Cor Inc., Lincoln, NE). For each VIGS experiment, 6th and 7th fully expanded leaves from 6 pTRV and pTRV‐gene plants were used to record all above parameters. The experiment was repeated twice with similar settings and parameters. The measurement conditions were as follows: leaf temperature: 27 °C, leaf‐air vapour pressure deficit: 1.5 ± 0.5 kPa, photosynthetic photon flux: 300 μmol/m^2^/s, relative air humidity: 70% and ambient CO_2_ concentration: 400 ± 5 μmol/mol. WUEi was calculated using the following formula: photosynthetic rate (A)/ transpiration rate (E).

### Histochemical detection of H_2_O_2_ and cell death assay

Histochemical detection of hydrogen peroxide (H_2_O_2_) was done using 3,3'‐diaminobenzidine (DAB) staining assay (Daudi *et al.*, 2012), and measurement of cell death was done using Trypan blue as described previously (Koch and Slusarenko, 1990). The 6th and 7th leaves from 6‐week‐old pTRV and pTRV‐gene plants were used immediately after HS (45 °C for 4.5 h) for the above assays. Experiments were repeated twice with similar parameters with sample size of six plants per replicate. The primers used in the study are listed in Table S5.

## Conflict of interest

The authors declare no conflict of interests.

## Author contributions

SM conceived the research project and supervised the experiments; SB, SR and SJ designed and performed most of the experiments and analysed the data; CB and JRD did functional promoter work; SB, SR, SJ and SM wrote the article; CB complemented the writing and figure preparation. SM agrees to serve as the author responsible for contact and ensures communication.

## Supporting information


**Figure S1** Comparative analysis of various tomato cultivars in response to HS.
**Figure S2** Transcriptome analysis of CLN and CA4 leaf under control and heat stress.
**Figure S** **3** The GO term enrichment analysis of gene set showing conserved up‐regulation in response to heat stress.
**Figure S4** The GO term enrichment analysis (biological process) of gene set showing conserved down‐regulation in response to heat stress.
**Figure S5** The GO term enrichment analysis (molecular function) of gene set showing conserved down‐regulation in response to heat stress.
**Figure S6** The GO term enrichment analysis (cellular component and protein class) of gene set showing conserved down‐regulation in response to heat stress.
**Figure S7** The GO term enrichment analysis of gene set showing tolerant cultivar specific HS response.
**Figure S8** The GO term enrichment analysis of gene set showing sensitive cultivar specific up‐regulation.
**Figure S9** The GO term enrichment analysis of gene set showing sensitive cultivar specific down‐regulation.
**Figure S10** Expression analysis of antagonistically selected genes in different tolerant and sensitive cultivars.
**Figure S11** Virus Induced Gene Silencing of *Acylsugar acyltransferase*, *Notabilis* and *Pin‐II type proteinase inhibitor 1 (PI‐II)*.
**Figure S12** *GUS:reporter assays of CLN and CA4 ASAT, Notabilis promoters in CLN and CA4 background.*
**Figure S13** TF families and cis‐elements associated with *Acylsugar acyltransferase (ASAT)* promoter.
**Figure S14** Average day/night temperature (°C) in field for the assessment of tomato cultivars for thermotolerance.

**Table S1** Summary of paired‐end dataset analysis.

**Table S2** The list and expression level (RPKM and fold change) of all the genes in the study.

**Table S3** KEGG pathway enrichment analysis of HS responsive genes in leaf of CLN and CA4.

**Table S4** The expression level of transcription factors in leaves of CLN and CA4 under control and HS conditions.

**Table S5** List of cultivars and primers used in the study.
