# Supplementary material for: Characterization of novel regulators for heat stress tolerance in tomato from Indian sub‐continent
Source: Plant Biotechnol J. 2020 Sep 1;18(10):2118–32. doi: 10.1111/pbi.13371 (PMC7540533; doi:10.1111/pbi.13371)
Supplement: Supplementary file 1 — Figure S1 Comparative analysis of various tomato cultivars in response to HS. Figure S2 Transcriptome analysis of CLN and CA4 leaf under control and heat stress. Figure S 3 The GO term enrichment analysis of gene set showing conserved up‐regulation in response to heat stress. Figure S4 The GO term enrichment analysis (biological process) of gene set showing conserved down‐regulation in response to heat stress. Figure S5 The GO term enrichment analysis (molecular function) of gene set showing conserved down‐regulation in response to heat stress. Figure S6 The GO term enrichment analysis (cellular component and protein class) of gene set showing conserved down‐regulation in response to heat stress. Figure S7 The GO term enrichment analysis of gene set showing tolerant cultivar specific HS response. Figure S8 The GO term enrichment analysis of gene set showing sensitive cultivar specific up‐regulation. Figure S9 The GO term enrichment analysis of gene set showing sensitive cultivar specific down‐regulation. Figure S10 Expression analysis of antagonistically selected genes in different tolerant and sensitive cultivars. Figure S11 Virus Induced Gene Silencing of Acylsugar acyltransferase, Notabilis and Pin‐II type proteinase inhibitor 1 (PI‐II). Figure S12 GUS:reporter assays of CLN and CA4 ASAT, Notabilis promoters in CLN and CA4 background. Figure S13 TF families and cis‐elements associated with Acylsugar acyltransferase (ASAT) promoter. Figure S14 Average day/night temperature (°C) in field for the assessment of tomato cultivars for thermotolerance. [file PBI-18-2118-s005.pdf]

Characterization of novel regulators for heat stress tolerance in tomato from Indian sub-continent

Sonia Balyan[1], Sombir Rao[1], Sarita Jha[1], Chandni Bansal, Jaishri Rubina Das and Saloni Mathur*

Supplementary Data

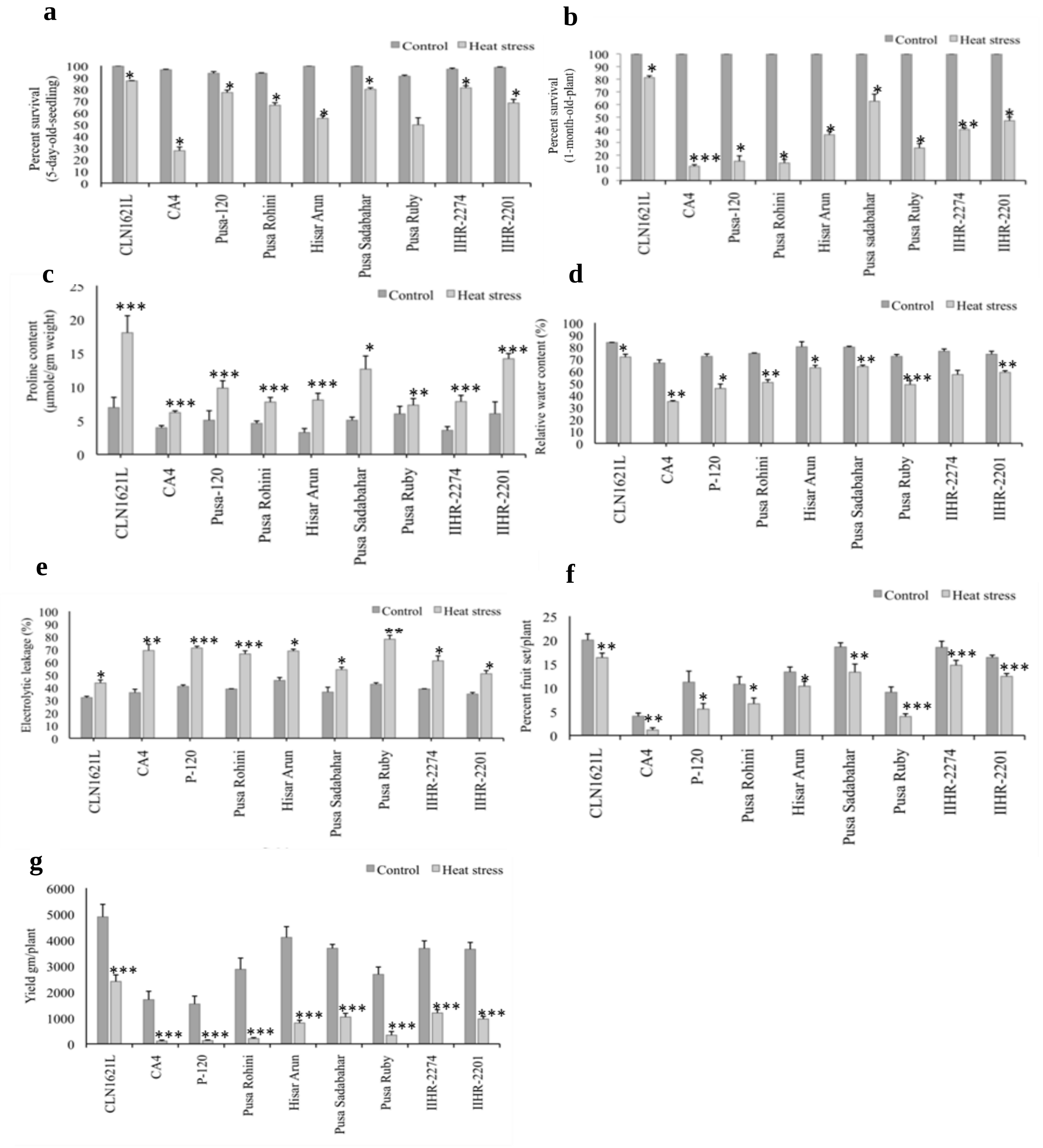

Figure S1. Comparative analysis of various tomato cultivars in response to HS. (a-b) The survival assays were performed at 5-days-old seedlings (a) and 1-month-old plants (b) in response to heat stress after overnight recovery. The seedlings were exposed to heat stress at 45ºC for 4.5 h. The 1-month-old plants were acclimated by gradual increasing temperatures from 26ºC to 45ºC for 4h followed by heat stress at 45ºC for 4.5h. (c-e) The measurements of proline content, relative water content and electrolytic leakage in leaves of 1-month-old plants of different cultivars under heat stress. (f-g) The effect of heat stress on percentage fruit set and tomato yield of different cultivars. The data was collected for two consecutive years in normal tomato growing season (October to February; 2014-2015 and 2015-2016) and warm season (February to June; 2015 and 2016). The error bars depict standard error. *p<0.05, **p<0.01 and ***p<0.001.

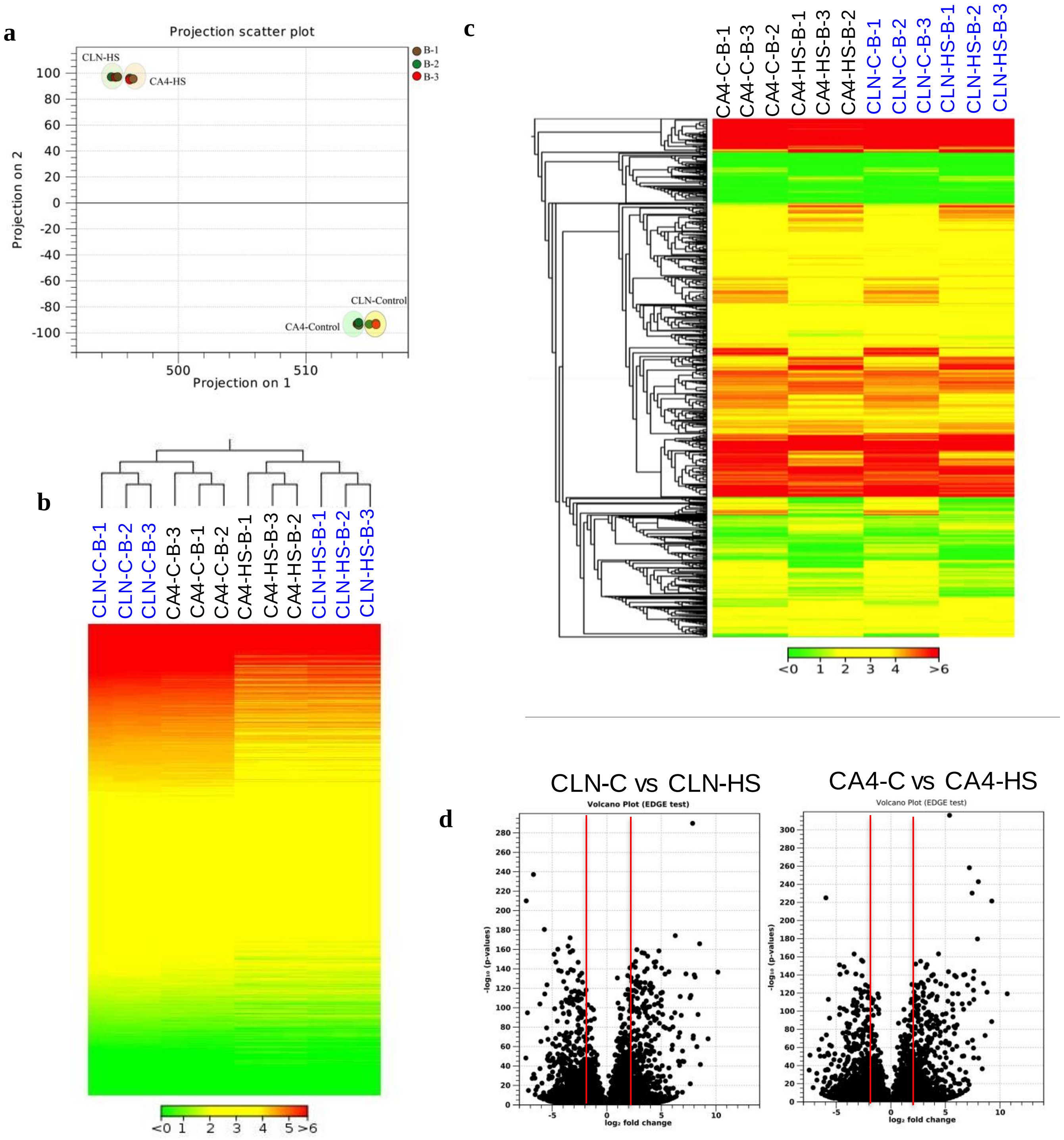

Figure S2. **Transcriptome analysis of CLN and CA4 leaf under control and heat stress. (a)** PCA plots of all RNA-seq libraries of CLN and CA4 under control and heat stress regimens. **(b and c)** Hierarchical clustering of different samples **(b)** and genes **(c)** in response to heat stress in CLN and CA4. The heat maps were plotted using the $\log_2$ transformed RPKM values following the average clustering algorithm using CLC Genomics Workbench software. **(d)** Volcano plots of all the expressed genes in CLN-C vs. CLN-H and CA4-C vs. CA4-H wherein the X and Y axis represents the fold change and $-\log_{10}$ (FDR p-value), respectively.

a

GO biological process complete	NUMBER OF GENES	FOLD ENRICHMENT	FDR P-VALUE
cellular response to nutrient levels (GO:0031669)	6	24	0.001
cellular response to phosphate starvation (GO:0016036)	6	24	0.001
cellular response to starvation (GO:0009267)	6	24	0.001
protein refolding (GO:0042026)	6	24	0.001
response to starvation (GO:0042594)	6	24	0.001
response to nutrient levels (GO:0031667)	6	21	0.001
cellular response to external stimulus (GO:0071496)	6	16	0.003
cellular response to extracellular stimulus (GO:0031668)	6	16	0.003
response to extracellular stimulus (GO:0009991)	6	15	0.003
protein folding (GO:0006457)	20	9	0.000

b

GO molecular function complete	NUMBER OF GENES	FOLD ENRICHMENT	FDR P-VALUE
3'-5'-exoribonuclease activity (GO:0000175)	4	14	0.038
3'-5' exonuclease activity (GO:0008408)	6	7	0.034
unfolded protein binding (GO:0051082)	8	6	0.016
RNA binding (GO:0003723)	30	2	0.021

c

PANTHER Protein Class	NUMBER OF GENES	FOLD ENRICHMENT	FDR P-VALUE
chaperone (PC00072)	30	7	0.000
winged helix/forkhead transcription factor (PC00246)	7	5	0.017
translation elongation factor (PC00222)	8	5	0.017
translation factor (PC00223)	16	3	0.008
mRNA splicing factor (PC00148)	18	3	0.008
mRNA processing factor (PC00147)	20	2	0.016

Figure S3. The GO term enrichment analysis of gene set showing conserved up-regulation in response to heat stress. The table showing enrichment analysis for biological process **(a),** molecular function **(b)** and protein class **(c)** (fold enrichment ≥2 and p-value ≤0.05) in the genes showing up-regulation in both (CLN and CA4) in response to heat stress.

GO biological process complete	NUMBER OF GENES	FOLD ENRICHMENT	FDR P-VALUE
small GTPase mediated signal transduction (GO:0007264)	5	11	0.010
water transport (GO:0006833)	5	9.95	0.012
fluid transport (GO:0042044)	5	9.95	0.012
xyloglucan metabolic process (GO:0010411)	7	7.66	0.004
tropism (GO:0009606)	7	7.66	0.004
steroid biosynthetic process (GO:0006694)	8	7.3	0.002
DNA replication (GO:0006260)	14	6.81	0.000
cellular response to auxin stimulus (GO:0071365)	14	6.81	0.000
auxin-activated signaling pathway (GO:0009734)	14	6.81	0.000
steroid metabolic process (GO:0008202)	8	6.74	0.003
anatomical structure morphogenesis (GO:0009653)	7	6.66	0.008
serine family amino acid metabolic process (GO:0009069)	7	6.13	0.011
hemicellulose metabolic process (GO:0010410)	7	6.13	0.011
cell wall polysaccharide metabolic process (GO:0010383)	7	6.13	0.011
organic acid catabolic process (GO:0016054)	7	5.89	0.012
carboxylic acid catabolic process (GO:0046395)	7	5.89	0.012
shoot system development (GO:0048367)	7	5.47	0.017
DNA conformation change (GO:0071103)	6	5.47	0.037
regulation of hormone levels (GO:0010817)	8	5.31	0.010
photosynthesis, light harvesting (GO:0009765)	6	5.25	0.042
plant organ development (GO:0099402)	7	5.11	0.023
terpenoid metabolic process (GO:0006721)	9	5.05	0.007
fatty acid biosynthetic process (GO:0006633)	14	5.02	0.000
cell wall biogenesis (GO:0042546)	7	4.94	0.026
response to radiation (GO:0009314)	9	4.93	0.008
response to light stimulus (GO:0009416)	9	4.93	0.008
monocarboxylic acid biosynthetic process (GO:0072330)	18	4.81	0.000
multicellular organism development (GO:0007275)	20	4.61	0.000
hormone-mediated signaling pathway (GO:0009755)	16	4.61	0.000
cellular response to hormone stimulus (GO:0032870)	16	4.55	0.000
cellular response to endogenous stimulus (GO:0071495)	16	4.55	0.000
isoprenoid metabolic process (GO:0006720)	12	4.53	0.002
terpenoid biosynthetic process (GO:0016114)	7	4.51	0.040
anatomical structure development (GO:0048856)	23	4.5	0.000
fatty acid metabolic process (GO:0006631)	14	4.44	0.001
cellular response to organic substance (GO:0071310)	16	4.43	0.000
developmental process (GO:0032502)	24	4.41	0.000
glucan metabolic process (GO:0044042)	13	4.31	0.001
cellular glucan metabolic process (GO:0006073)	13	4.31	0.001
isoprenoid biosynthetic process (GO:0008299)	10	4.29	0.009
system development (GO:0048731)	14	4.26	0.001
lipid biosynthetic process (GO:0008610)	34	4.25	0.000
organic acid biosynthetic process (GO:0016053)	33	4.22	0.000
carboxylic acid biosynthetic process (GO:0046394)	33	4.22	0.000
response to external stimulus (GO:0009605)	13	4.07	0.002
cellular polysaccharide metabolic process (GO:0044264)	13	4.01	0.003
response to abiotic stimulus (GO:0009628)	18	3.94	0.000
reproductive system development (GO:0061458)	9	3.79	0.029
reproductive structure development (GO:0048608)	9	3.79	0.029
multicellular organismal process (GO:0032501)	21	3.74	0.000
monocarboxylic acid metabolic process (GO:0032787)	24	3.6	0.000
developmental process involved in reproduction (GO:0003006)	9	3.58	0.040
small molecule biosynthetic process (GO:0044283)	36	3.55	0.000
alpha-amino acid metabolic process (GO:1901605)	18	3.46	0.001
external encapsulating structure organization (GO:0045229)	23	3.45	0.000
carboxylic acid metabolic process (GO:0019752)	52	3.39	0.000
lipid metabolic process (GO:0006629)	47	3.38	0.000
oxoacid metabolic process (GO:0043436)	52	3.36	0.000
organic acid metabolic process (GO:0006082)	52	3.34	0.000
cell wall organization (GO:0071555)	22	3.34	0.000
cellular amino acid biosynthetic process (GO:0008652)	14	3.33	0.007
intracellular signal transduction (GO:0035556)	13	3.27	0.011
signal transduction (GO:0007165)	38	3.25	0.000
cellular amino acid metabolic process (GO:0006520)	27	3.25	0.000
signaling (GO:0023052)	38	3.24	0.000
cellular lipid metabolic process (GO:0044255)	30	3.24	0.000
polysaccharide metabolic process (GO:0005976)	20	3.2	0.001
DNA metabolic process (GO:0006259)	14	3.16	0.010
cellular carbohydrate metabolic process (GO:0044262)	15	2.93	0.012
cell communication (GO:0007154)	38	2.93	0.000
photosynthesis (GO:0015979)	18	2.92	0.005
negative regulation of molecular function (GO:0044092)	14	2.92	0.017
negative regulation of catalytic activity (GO:0043086)	14	2.92	0.017
defense response (GO:0006952)	13	2.9	0.026
response to auxin (GO:0009733)	17	2.88	0.007
cell wall organization or biogenesis (GO:0071554)	22	2.87	0.001
drug catabolic process (GO:0042737)	16	2.74	0.015
small molecule metabolic process (GO:0044281)	64	2.55	0.000
response to hormone (GO:0009725)	21	2.48	0.011
response to endogenous stimulus (GO:0009719)	21	2.48	0.011
carbohydrate metabolic process (GO:0005975)	48	2.48	0.000
organic substance biosynthetic process (GO:1901576)	113	2.46	0.000
cellular response to stimulus (GO:0051716)	51	2.45	0.000
regulation of catalytic activity (GO:0050790)	19	2.43	0.022
response to organic substance (GO:0010033)	21	2.41	0.013
regulation of molecular function (GO:0065009)	19	2.4	0.023
drug metabolic process (GO:0017144)	24	2.4	0.007
biosynthetic process (GO:0009058)	117	2.39	0.000
cellular biosynthetic process (GO:0044249)	106	2.38	0.000
cellular response to chemical stimulus (GO:0070887)	21	2.35	0.016
response to stimulus (GO:0050896)	79	2.34	0.000
regulation of biological quality (GO:0065008)	20	2.33	0.022
transmembrane transport (GO:0055085)	50	2.28	0.000
cellular component organization (GO:0016043)	46	2.27	0.000
organic cyclic compound biosynthetic process (GO:1901362)	31	2.21	0.004
response to stress (GO:0006950)	32	2.16	0.004
cellular component organization or biogenesis (GO:0071840)	47	2.13	0.000
proteolysis (GO:0006508)	28	2.12	0.012
oxidation-reduction process (GO:0055114)	94	2.12	0.000
transport (GO:0006810)	80	2.1	0.000
establishment of localization (GO:0051234)	80	2.09	0.000
localization (GO:0051179)	80	2.07	0.000

Figure S4. The GO term enrichment analysis (biological process) of gene set showing conserved down-regulation in response to heat stress. The table showing enrichment analysis for biological process (fold enrichment ≥2 and p-value ≤0.05) in the genes showing down-regulation in both (CLN and CA4) in response to heat stress.

GO molecular function complete	NUMBER OF GENES	FOLD ENRICHMENT	FDR P-VALUE
xyloglucan:xyloglucosyl transferase activity (GO:0016762)	7	7.300	0.007
oxidoreductase activity, acting on the CH-CH group of donors, NAD or NADP as acceptor (GO:0016628	5	6.840	0.037
transaminase activity (GO:0008483)	7	6.130	0.012
transferase activity, transferring nitrogenous groups (GO:0016769)	7	6.130	0.012
oxidoreductase activity, acting on CH-OH group of donors (GO:0016614)	14	5.280	0.000
oxidoreductase activity, acting on the aldehyde or oxo group of donors, NAD or NADP as acceptor (GO:	7	5.110	0.023
protein heterodimerization activity (GO:0046982)	12	4.860	0.002
NAD binding (GO:0051287)	9	4.810	0.010
oxidoreductase activity, acting on the CH-OH group of donors, NAD or NADP as acceptor (GO:001661	10	4.470	0.008
oxidoreductase activity, acting on the CH-CH group of donors (GO:0016627)	9	4.380	0.014
catalytic activity, acting on DNA (GO:0140097)	7	4.260	0.048
channel activity (GO:0015267)	13	4.180	0.002
passive transmembrane transporter activity (GO:0022803)	13	4.180	0.003
aspartic-type endopeptidase activity (GO:0004190)	11	4.080	0.009
aspartic-type peptidase activity (GO:0070001)	11	4.080	0.008
glucosyltransferase activity (GO:0046527)	10	4.050	0.012
carboxylic ester hydrolase activity (GO:0052689)	9	3.580	0.038
coenzyme binding (GO:0050662)	36	3.200	0.000
endopeptidase activity (GO:0004175)	22	3.110	0.001
lipid binding (GO:0008289)	11	3.090	0.037
carbohydrate binding (GO:0030246)	12	3.020	0.029
enzyme inhibitor activity (GO:0004857)	14	3.000	0.015
transferase activity, transferring acyl groups (GO:0016746)	22	2.990	0.001
GTPase activity (GO:0003924)	13	2.930	0.024
transferase activity, transferring acyl groups other than amino-acyl groups (GO:0016747)	17	2.800	0.011
enzyme regulator activity (GO:0030234)	19	2.740	0.008
hydrolase activity, hydrolyzing O-glycosyl compounds (GO:0004553)	23	2.710	0.002
molecular function regulator (GO:0098772)	21	2.690	0.005
GTP binding (GO:0005525)	16	2.610	0.022
guanyl nucleotide binding (GO:0019001)	16	2.610	0.022
guanyl ribonucleotide binding (GO:0032561)	16	2.610	0.023
purine nucleoside binding (GO:0001883)	16	2.610	0.023
purine ribonucleoside binding (GO:0032550)	16	2.610	0.022
nucleoside binding (GO:0001882)	17	2.600	0.018
phosphoric ester hydrolase activity (GO:0042578)	17	2.570	0.020
hydrolase activity, acting on glycosyl bonds (GO:0016798)	23	2.530	0.007
hydrolase activity, acting on ester bonds (GO:0016788)	38	2.510	0.000
ribonucleoside binding (GO:0032549)	16	2.470	0.049
peptidase activity, acting on L-amino acid peptides (GO:0070011)	26	2.450	0.004
peptidase activity (GO:0008233)	26	2.360	0.006
nucleoside-triphosphatase activity (GO:0017111)	24	2.350	0.010
transporter activity (GO:0005215)	45	2.310	0.000
pyrophosphatase activity (GO:0016462)	25	2.300	0.011
hydrolase activity (GO:0016787)	124	2.280	0.000
hydrolase activity, acting on acid anhydrides, in phosphorus-containing anhydrides (GO:0016818)	25	2.280	0.012
transmembrane transporter activity (GO:0022857)	43	2.280	0.000
hydrolase activity, acting on acid anhydrides (GO:0016817)	25	2.260	0.012
cofactor binding (GO:0048037)	60	2.210	0.000
oxidoreductase activity (GO:0016491)	89	2.160	0.000

Figure S5. The GO term enrichment analysis (molecular function) of gene set showing conserved down-regulation in response to heat stress. The table showing enrichment analysis for molecular function (fold enrichment ≥2 and p-value ≤0.05) in the genes showing down-regulation in both (CLN and CA4) in response to heat stress.

a

GO cellular component complete	NUMBER OF GENES	FOLD ENRICHMENT	FDR P-VALUE
DNA packaging complex (GO:0044815)	12	6.74	0.000
nucleosome (GO:0000786)	12	6.74	0.000
protein-DNA complex (GO:0032993)	12	6.41	0.000
chloroplast stroma (GO:0009570)	6	5.71	0.017
plastid stroma (GO:0009532)	6	5.71	0.018
chromatin (GO:0000785)	12	4.86	0.001
photosystem I (GO:0009522)	9	4.38	0.008
photosystem II (GO:0009523)	11	4.01	0.004
chromosomal part (GO:0044427)	13	3.6	0.003
photosystem (GO:0009521)	14	3.48	0.003
apoplast (GO:0048046)	11	3.44	0.011
chromosome (GO:0005694)	14	3.33	0.004
thylakoid membrane (GO:0042651)	14	2.98	0.009
photosynthetic membrane (GO:0034357)	17	2.95	0.003
cell wall (GO:0005618)	15	2.88	0.008
external encapsulating structure (GO:0030312)	15	2.88	0.008
thylakoid part (GO:0044436)	17	2.8	0.005
chloroplast part (GO:0044434)	16	2.69	0.010
plastid part (GO:0044435)	16	2.67	0.010
thylakoid (GO:0009579)	17	2.64	0.009
extracellular region (GO:0005576)	28	2.35	0.003
membrane protein complex (GO:0098796)	19	2.19	0.038
cell periphery (GO:0071944)	25	2.15	0.012
intracellular non-membrane-bounded organelle (GO:0043232)	41	2.04	0.002
non-membrane-bounded organelle (GO:0043228)	41	2.04	0.002

b

PANTHER Protein Class	NUMBER OF GENES	FOLD ENRICHMENT	FDR P-VALUE
carbohydrate phosphatase (PC00066)	4	11	0.017
histone (PC00118)	8	4.73	0.010
small GTPase (PC00208)	9	4.19	0.010
transaminase (PC00216)	9	3.46	0.026
dehydratase (PC00091)	15	2.76	0.010
G-protein (PC00020)	12	2.58	0.040
serine protease (PC00203)	44	2.54	0.000
aspartic protease (PC00053)	12	2.53	0.044
epimerase/racemase (PC00096)	16	2.43	0.028
lyase (PC00144)	30	2.4	0.001
isomerase (PC00135)	22	2.3	0.010
protease (PC00190)	73	2	0.000

Figure S6. The GO term enrichment analysis (cellular component and protein class) of gene set showing conserved down-regulation in response to heat stress. The tables showing enrichment analysis for cellular component **(a)** and protein class **(b)** (fold enrichment ≥2 and p-value ≤0.05) in the genes showing down-regulation in both (CLN and CA4) in response to heat stress.

a

GO biological process complete	NUMBER OF GENES	FOLD ENRICHMENT	FDR P-VALUE
photosynthesis, light harvesting (GO:0009765)	8	56.29	0.0000
protein-chromophore linkage (GO:0018298)	8	33.51	0.0000
photosynthesis, light reaction (GO:0019684)	8	21.32	0.0000
photosynthesis (GO:0015979)	15	19.55	0.0000
generation of precursor metabolites and energy (GO:0006091)	10	6.93	0.0003
translation (GO:0006412)	11	6.39	0.0002
peptide biosynthetic process (GO:0043043)	11	6.3	0.0003
peptide metabolic process (GO:0006518)	11	6.12	0.0003
amide biosynthetic process (GO:0043604)	11	5.94	0.0004
cellular amide metabolic process (GO:0043603)	11	5.45	0.0008
organonitrogen compound biosynthetic process (GO:1901566)	18	5.34	0.0000
cellular nitrogen compound biosynthetic process (GO:0044271)	15	4.61	0.0002
cellular macromolecule biosynthetic process (GO:0034645)	12	3.97	0.0063
macromolecule biosynthetic process (GO:0009059)	12	3.85	0.0080
cellular biosynthetic process (GO:0044249)	20	3.6	0.0002
organic substance biosynthetic process (GO:1901576)	20	3.5	0.0002
cellular protein metabolic process (GO:0044267)	26	3.47	0.0000
biosynthetic process (GO:0009058)	21	3.45	0.0002
protein metabolic process (GO:0019538)	29	3.31	0.0000
organonitrogen compound metabolic process (GO:1901564)	36	3.29	0.0000
cellular nitrogen compound metabolic process (GO:0034641)	16	2.88	0.0153
cellular macromolecule metabolic process (GO:0044260)	26	2.8	0.0003
nitrogen compound metabolic process (GO:0006807)	36	2.69	0.0000
cellular metabolic process (GO:0044237)	44	2.66	0.0000
metabolic process (GO:0008152)	59	2.57	0.0000
cellular process (GO:0009987)	52	2.56	0.0000
organic substance metabolic process (GO:0071704)	44	2.52	0.0000
primary metabolic process (GO:0044238)	41	2.49	0.0000
macromolecule metabolic process (GO:0043170)	29	2.43	0.0013
biological_process (GO:0008150)	74	2.07	0.0000

b

GO molecular function complete	NUMBER OF GENES	FOLD ENRICHMENT	FDR P-VALUE
glycopeptide alpha-N-acetylgalactosaminidase activity (GO:0033926)	2	> 100	0.0326
catalytic activity, acting on a glycoprotein (GO:0140103)	2	87.96	0.0454
cysteine synthase activity (GO:0004124)	2	87.96	0.0426
chlorophyll binding (GO:0016168)	8	38.04	0.0000
ATPase activity, coupled to transmembrane movement of ions, rotationa	3	31.04	0.0249
ATPase-coupled cation transmembrane transporter activity (GO:001982	4	25.13	0.0065
ATPase-coupled ion transmembrane transporter activity (GO:0042625)	4	25.13	0.0057
ATPase-coupled transmembrane transporter activity (GO:0042626)	4	16.36	0.0212
primary active transmembrane transporter activity (GO:0015399)	4	14.66	0.0242
structural constituent of ribosome (GO:0003735)	11	8.6	0.0000
structural molecule activity (GO:0005198)	11	7.47	0.0001
tetrapyrrole binding (GO:0046906)	10	6.47	0.0013
cofactor binding (GO:0048037)	13	3.86	0.0073

c

GO cellular component complete	NUMBER OF GENES	FOLD ENRICHMENT	FDR P-VALUE
photosystem I reaction center (GO:0009538)	3	58.64	0.0005
photosystem I (GO:0009522)	10	39.09	0.0000
proton-transporting two-sector ATPase complex, catalytic domain (GO:0	2	29.32	0.0364
photosystem II (GO:0009523)	9	26.39	0.0000
photosystem (GO:0009521)	13	25.99	0.0000
chloroplast thylakoid membrane (GO:0009535)	10	20.22	0.0000
plastid thylakoid membrane (GO:0055035)	10	20.22	0.0000
photosynthetic membrane (GO:0034357)	14	19.55	0.0000
organelle subcompartment (GO:0031984)	11	19.16	0.0000
thylakoid membrane (GO:0042651)	11	18.79	0.0000
thylakoid part (GO:0044436)	14	18.52	0.0000
chloroplast thylakoid (GO:0009534)	10	18.32	0.0000
plastid thylakoid (GO:0031976)	10	18.32	0.0000
thylakoid (GO:0009579)	14	17.47	0.0000
membrane protein complex (GO:0098796)	15	13.89	0.0000
chloroplast part (GO:0044434)	10	13.53	0.0000
plastid part (GO:0044435)	10	13.43	0.0000
chloroplast (GO:0009507)	14	8.76	0.0000
plastid (GO:0009536)	14	8.61	0.0000
ribosome (GO:0005840)	11	8.38	0.0000
protein-containing complex (GO:0032991)	32	6.95	0.0000
ribonucleoprotein complex (GO:1990904)	11	6.54	0.0000
cytoplasmic part (GO:0044444)	31	5.65	0.0000
intracellular non-membrane-bounded organelle (GO:0043232)	13	5.2	0.0000
non-membrane-bounded organelle (GO:0043228)	13	5.2	0.0000
cytoplasm (GO:0005737)	33	4.94	0.0000
intracellular organelle part (GO:0044446)	15	3.74	0.0003
organelle part (GO:0044422)	15	3.74	0.0003
intracellular (GO:0005622)	44	3.29	0.0000
intracellular part (GO:0044424)	44	3.29	0.0000
cell part (GO:0044464)	46	3.17	0.0000
intracellular organelle (GO:0043229)	36	3.1	0.0000
organelle (GO:0043226)	36	3.1	0.0000
cell (GO:0005623)	47	3.09	0.0000
intracellular membrane-bounded organelle (GO:0043231)	25	2.57	0.0003
membrane-bounded organelle (GO:0043227)	25	2.53	0.0003
cellular_component (GO:0005575)	64	2.17	0.0000
membrane (GO:0016020)	35	2.08	0.0005

Figure S7. The GO term enrichment analysis of gene set showing tolerant cultivar specific HS response. The tables showing enrichment analysis for biological process **(a),** molecular function **(b)** and cellular component **(c)** for genes exhibiting down-regulation in response to HS only in CLN and not in CA4. The genes with significant down-regulation (≥2 FC and p-value ≤0.05 in CLN; ≤2 FC in CA4) were considered.

GO biological process complete	NUMBER OF GENES	FOLD ENRICHMENT	FDR P-VALUE
translation (GO:0006412)	12	6.86	0.0004
peptide biosynthetic process (GO:0043043)	12	6.77	0.0003
peptide metabolic process (GO:0006518)	12	6.58	0.0003
amide biosynthetic process (GO:0043604)	12	6.38	0.0004
cellular amide metabolic process (GO:0043603)	12	5.86	0.0006
gene expression (GO:0010467)	17	5.01	0.0002
cellular macromolecule biosynthetic process (GO:0034645)	13	4.23	0.0034
cellular nitrogen compound biosynthetic process (GO:0044271)	14	4.23	0.0019
macromolecule biosynthetic process (GO:0009059)	13	4.11	0.0040
organonitrogen compound biosynthetic process (GO:1901566)	14	4.09	0.0025
cellular nitrogen compound metabolic process (GO:0034641)	20	3.55	0.0006
biosynthetic process (GO:0009058)	20	3.23	0.0014
cellular biosynthetic process (GO:0044249)	18	3.19	0.0034
organic substance biosynthetic process (GO:1901576)	18	3.11	0.0043
cellular protein metabolic process (GO:0044267)	19	2.5	0.0312
protein metabolic process (GO:0019538)	21	2.36	0.0393
nitrogen compound metabolic process (GO:0006807)	32	2.35	0.0016
organonitrogen compound metabolic process (GO:1901564)	26	2.34	0.0087
macromolecule metabolic process (GO:0043170)	28	2.31	0.0058
organic substance metabolic process (GO:0071704)	36	2.03	0.0065
primary metabolic process (GO:0044238)	34	2.03	0.0112
cellular metabolic process (GO:0044237)	34	2.02	0.0111
**GO molecular function complete**			
structural constituent of ribosome (GO:0003735)	11	8.47	0.0002
structural molecule activity (GO:0005198)	11	7.36	0.0004
**GO cellular component complete**			
ribosome (GO:0005840)	12	9	0.0000
ribonucleoprotein complex (GO:1990904)	13	7.61	0.0000
intracellular non-membrane-bounded organelle (GO:0043232)	13	5.12	0.0003
non-membrane-bounded organelle (GO:0043228)	13	5.12	0.0002
protein-containing complex (GO:0032991)	19	4.06	0.0001
cytoplasmic part (GO:0044444)	21	3.77	0.0001
cytoplasm (GO:0005737)	21	3.09	0.0004
intracellular organelle (GO:0043229)	30	2.54	0.0002
organelle (GO:0043226)	30	2.54	0.0002
intracellular (GO:0005622)	31	2.29	0.0008
intracellular part (GO:0044424)	31	2.29	0.0007
cell part (GO:0044464)	33	2.24	0.0008
cell (GO:0005623)	34	2.2	0.0008

Figure S8. The GO term enrichment analysis of gene set showing sensitive cultivar specific upregulation. The tables showing enrichment analysis for biological process, molecular component and protein class for genes exhibiting upregulation in response to HS only in CA4 and not in CLN (≥2 FC and p-value ≤0.05 in CA4; ≤2 FC in CLN).

GO biological process complete	NUMBER OF GENES	FOLD ENRICHMENT	FDR P-VALUE
second-messenger-mediated signaling (GO:0019932)	2	75.79	0.0461
calcium-mediated signaling (GO:0019722)	2	75.79	0.0449
monocarboxylic acid metabolic process (GO:0032787)	6	6.23	0.0414
cellular lipid metabolic process (GO:0044255)	7	5.23	0.0409
lipid metabolic process (GO:0006629)	10	4.99	0.0138
carbohydrate metabolic process (GO:0005975)	11	3.94	0.0235
small molecule metabolic process (GO:0044281)	14	3.87	0.0090
transmembrane transport (GO:0055085)	11	3.47	0.0374
protein phosphorylation (GO:0006468)	12	3.33	0.0309
regulation of transcription, DNA-templated (GO:0006355)	14	3.24	0.0245
regulation of gene expression (GO:0010468)	15	3.21	0.0179
regulation of RNA biosynthetic process (GO:2001141)	14	3.21	0.0243
regulation of nucleic acid-templated transcription (GO:1903506)	14	3.21	0.0230
regulation of RNA metabolic process (GO:0051252)	14	3.19	0.0229
regulation of nucleobase-containing compound metabolic process (GO:0019219)	14	3.17	0.0221
regulation of nitrogen compound metabolic process (GO:0051171)	16	3.17	0.0142
regulation of macromolecule metabolic process (GO:0060255)	17	3.17	0.0128
regulation of primary metabolic process (GO:0080090)	16	3.16	0.0137
regulation of cellular macromolecule biosynthetic process (GO:2000112)	14	3.13	0.0238
phosphorylation (GO:0016310)	14	3.12	0.0242
regulation of metabolic process (GO:0019222)	17	3.11	0.0126
regulation of macromolecule biosynthetic process (GO:0010556)	14	3.1	0.0242
regulation of cellular biosynthetic process (GO:0031326)	14	3.09	0.0240
regulation of biosynthetic process (GO:0009889)	14	3.09	0.0231
regulation of cellular metabolic process (GO:0031323)	15	2.89	0.0261
regulation of biological process (GO:0050789)	21	2.67	0.0133
phosphate-containing compound metabolic process (GO:0006796)	16	2.59	0.0458
regulation of cellular process (GO:0050794)	19	2.59	0.0230
phosphorus metabolic process (GO:0006793)	16	2.59	0.0454
biological regulation (GO:0065007)	22	2.48	0.0186
biological_process (GO:0008150)	94	2.27	0.0000
primary metabolic process (GO:0044238)	43	2.25	0.0003
organic substance metabolic process (GO:0071704)	44	2.17	0.0004
metabolic process (GO:0008152)	57	2.14	0.0000
cellular process (GO:0009987)	50	2.12	0.0002
**GO molecular function complete**			
GTPase activity (GO:0003924)	7	10.94	0.0005
proton transmembrane transporter activity (GO:0015078)	4	9.19	0.0426
purine nucleoside binding (GO:0001883)	7	7.92	0.0033
guanyl ribonucleotide binding (GO:0032561)	7	7.92	0.0031
purine ribonucleoside binding (GO:0032550)	7	7.92	0.0029
GTP binding (GO:0005525)	7	7.92	0.0028
guanyl nucleotide binding (GO:0019001)	7	7.92	0.0027
calcium ion binding (GO:0005509)	7	7.8	0.0026
ribonucleoside binding (GO:0032549)	7	7.47	0.0032
nucleoside binding (GO:0001882)	7	7.42	0.0033
cation transmembrane transporter activity (GO:0008324)	6	5.94	0.0245
pyrophosphatase activity (GO:0016462)	9	5.73	0.0032
hydrolase activity, acting on acid anhydrides, in phosphorus-containing anhydrides (GO:0016818)	9	5.68	0.0026
hydrolase activity, acting on acid anhydrides (GO:0016817)	9	5.64	0.0026
nucleoside-triphosphatase activity (GO:0017111)	8	5.41	0.0072
transmembrane transporter activity (GO:0022857)	11	4.04	0.0060
transporter activity (GO:0005215)	11	3.91	0.0071
protein kinase activity (GO:0004672)	12	3.39	0.0122
phosphotransferase activity, alcohol group as acceptor (GO:0016773)	13	3.23	0.0112
anion binding (GO:0043168)	31	3.13	0.0000
purine ribonucleoside triphosphate binding (GO:0035639)	24	3.11	0.0002
small molecule binding (GO:0036094)	31	3.09	0.0000
kinase activity (GO:0016301)	13	3.05	0.0173
ribonucleotide binding (GO:0032553)	25	2.98	0.0002
carbohydrate derivative binding (GO:0097367)	25	2.94	0.0002
nucleotide binding (GO:0000166)	27	2.92	0.0001
nucleoside phosphate binding (GO:1901265)	27	2.92	0.0001
purine ribonucleotide binding (GO:0032555)	24	2.9	0.0004
purine nucleotide binding (GO:0017076)	24	2.89	0.0004
transferase activity, transferring phosphorus-containing groups (GO:0016772)	13	2.72	0.0446
drug binding (GO:0008144)	20	2.69	0.0037
ion binding (GO:0043167)	46	2.63	0.0000
heterocyclic compound binding (GO:1901363)	51	2.57	0.0000
organic cyclic compound binding (GO:0097159)	51	2.57	0.0000
ATP binding (GO:0005524)	17	2.48	0.0244
transferase activity (GO:0016740)	24	2.37	0.0060
nucleic acid binding (GO:0003676)	21	2.36	0.0160
binding (GO:0005488)	67	2.33	0.0000
hydrolase activity (GO:0016787)	18	2.29	0.0491
molecular_function (GO:0003674)	100	2.14	0.0000
catalytic activity (GO:0003824)	26.68	2.1	0.0000
**GO cellular component complete**			
membrane part (GO:0044425)	39	2.09	0.0021
cell part (GO:0044464)	35	2.08	0.0042
membrane (GO:0016020)	40	2.04	0.0028

Figure S9. The GO term enrichment analysis of gene set showing sensitive cultivar specific down-regulation. The tables showing enrichment analysis for biological process **(a),** molecular component **(b)** and protein class **(c)** for genes exhibiting down-regulation in response to HS only in CA4 and not in CLN.

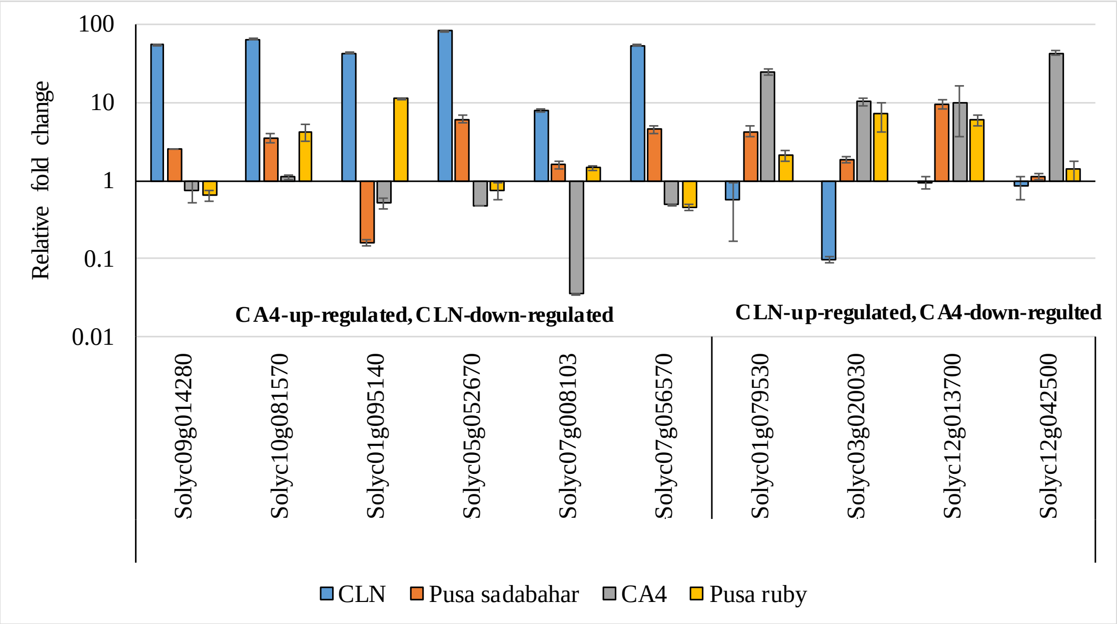

Figure S10. Expression analysis of antagonistically selected genes in different tolerant and sensitive cultivars. The qRT-PCR expression analysis of genes with inverse HS-response in contrasting cultivars in leaf of heat tolerant (CLN and Pusa Sadabahar) and sensitive (CA4 and Pusa Ruby) under heat stress conditions. For each condition two biological and three technical repeats were used. The expression levels of genes were calculated using the 2−ΔΔCt method and presented using fold change values transformed to $\log_2$ relative to respective Actin gene expression. The error bars represent standard error.

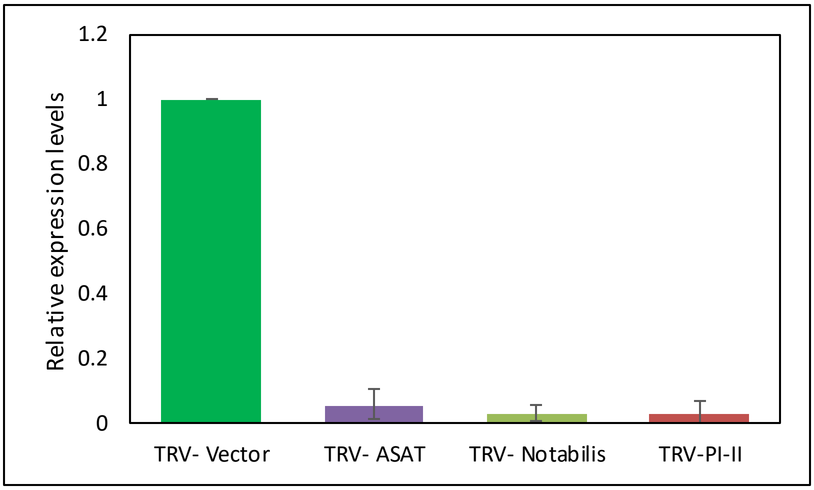

Figure S11. **Virus Induced Gene Silencing of *Acylsugar acyltransferase*, *Notabilis* and *Pin-II type proteinase inhibitor 1 (PI-II)*** Quantitative RT-PCR analysis of *ASAT*, *Notabilis* and PI-II genes in TRV and TRV-ASAT, TRV-*Notabilis* and TRV-*PI-II* plants confirming the silencing of their respective genes. The expression levels of genes were calculated using the 2−ΔΔCt method and presented using fold-change values transformed to $\log_2$ format compared with control.

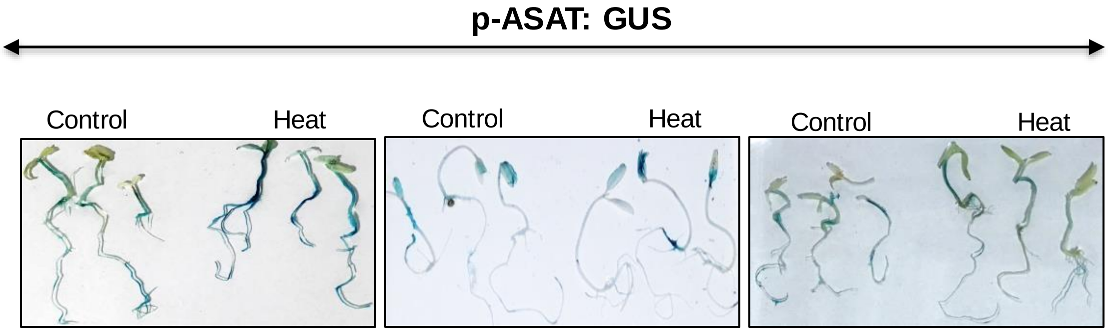

Promoter and genotype background	pCLN in CLN background	pCLN in CA4 background	pCA4 in CLN background
GUS fold change as per qRT-PCR	~2.5	Non differential	Non differential

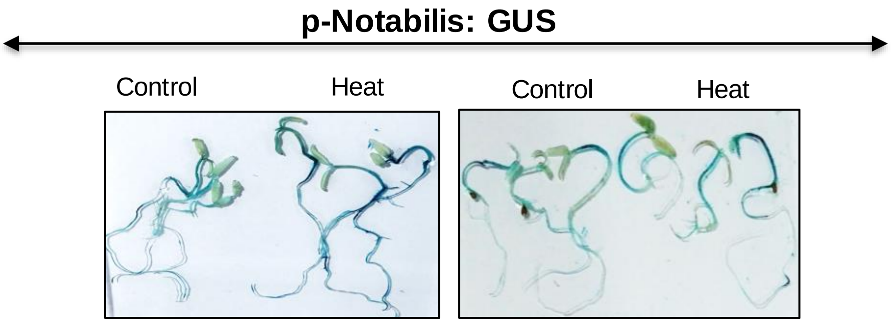

Promoter and genotype background	pCLN in CLN background	pCLN in CA4 background
GUS fold change as per qRT-PCR	~2.5	Non differential

Figure S12. ***GUS:reporter assays of CLN and CA4 ASAT, Notabilis promoters in CLN and CA4 background.*** Histochemical GUS expression patterns of pCLN/CA4-*ASAT:GUS* and pCLN-*Notabilis:GUS* reporter constructs upon transient infiltration in CLN and CA4 background after exposing to 0h (control) or 2h of heat stress at 45°C. The corresponding qRT-PCR fold change values of GUS transcripts is written below each set.

TF-family	No. of cis-element in ASAT promoter
AP2;ERF	2
bHLH	13
bZIP	5
C2H2	7
CSD	1
Dof	26
Tify	27
HB-TALE	7
HSF	4
Myb	3
NAC;NAM	1
SBP	3
Sox;YABBY	1
SRS	1
Trihelix	2
WRKY	4
ZF-HD	18

Figure S13. **TF families and cis-elements associated with *Acylsugar acyltransferase (ASAT)* promoter.** The number of various binding sites (cis-elements) of different transcription factors in the promoter of ASAT as predicted by PlantPAN database.

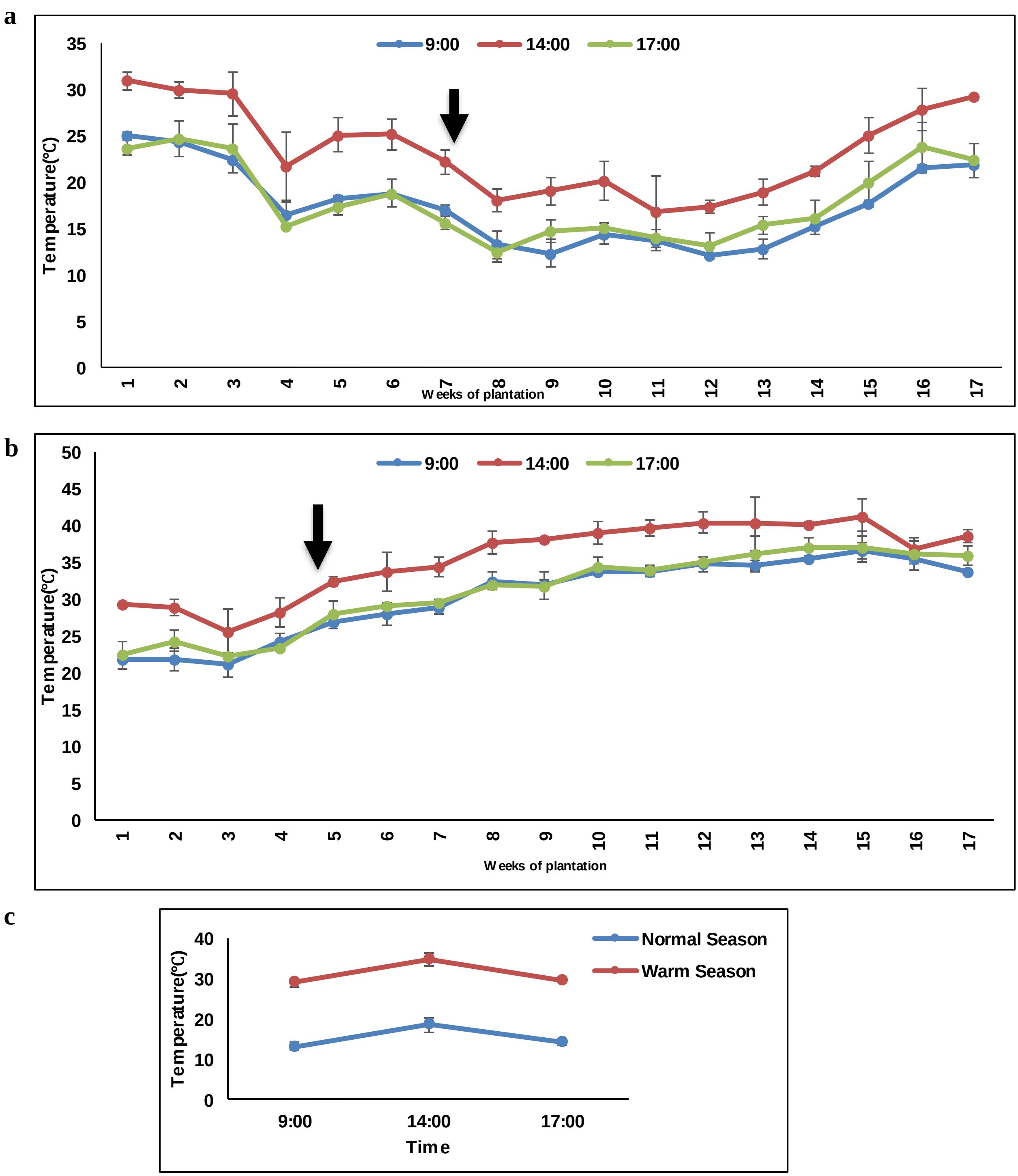

Figure S14. Average day/night temperature (℃) in field for the assessment of tomato cultivars for thermotolerance. Data recorded in year 2014-2016 during weeks of tomato cultivars plantation in experimental fields of National Institute of Plant Genome Research (NIPGR), New Delhi, India. **(a)** Normal Season average temperature (°C) (Week-1; 4th week of October 2014 and 2015). **(b)** Warm season average temperature (°C) (Week-1; 4th week of February 2015 and 2016). **(c)** The average day/night temperature (°C) during the flowering in normal season and warm season. Black arrows in '**a**' and '**b**' mark start time of flowering in tomato cultivars in both seasons.
