# Supplementary material for: Characterization of novel regulators for heat stress tolerance in tomato from Indian sub‐continent
Source: Plant Biotechnol J. 2020 Sep 1;18(10):2118–32. doi: 10.1111/pbi.13371 (PMC7540533; doi:10.1111/pbi.13371)
Supplement: Supplementary file 2 — Table S1 Summary of paired‐end dataset analysis. [file PBI-18-2118-s004.docx]

**Table S1**. **Summary of paired-end dataset analysis**. The tomato genome build 3.0 at SGN (Sol Genomics Networks) was used as the reference for mapping.

| **S.No.** | **Dataset** | **No. of trimmed good quality reads** | **No. of reads mapped in pair** | **Mapping percentage** |
| --- | --- | --- | --- | --- |
| 1 | CA4 control-A | 18938490 | 18373166 | 97.01 |
| 2 | CA4 control-B | 18938490 | 18380605 | 97.05 |
| 3 | CA4 control-C | 18938494 | 18399191 | 97.15 |
| 4 | CA4 heat stress-A | 18827392 | 17895037 | 95.5 |
| 5 | CA4 heat stress-B | 18827394 | 18052917 | 95.89 |
| 6 | CA4 heat stress-C | 18827392 | 18198840 | 96.66 |
| 7 | CLN control-A | 18957802 | 18378883 | 96.95 |
| 8 | CLN control-B | 18957803 | 18393516 | 97.02 |
| 9 | CLN control-C | 18957804 | 18415649 | 97.1 |
| 10 | CLN heat stress-A | 18419514 | 17812813 | 96.71 |
| 11 | CLN heat stress-B | 18419514 | 17833192 | 96.8 |
| 12 | CLN heat stress-C | 18419514 | 17855388 | 96.94 |
