# Supplementary material for: Characterization of novel regulators for heat stress tolerance in tomato from Indian sub‐continent
Source: Plant Biotechnol J. 2020 Sep 1;18(10):2118–32. doi: 10.1111/pbi.13371 (PMC7540533; doi:10.1111/pbi.13371)
Supplement: Supplementary file 6 — Table S5 List of cultivars and primers used in the study. [file PBI-18-2118-s001.docx]

**Table S5**: **List of cultivars and primers used in the study**

| 1. **Details about cultivar used:** | | |
| --- | --- | --- |
|  | *Cultivar* | *Name of Institute/Place procured from* |
|  | CLN1621L and CA4 | Asian Vegetable Research and Development Centre (AVRDC), Taiwan |
|  | Pusa-120, Pusa Rohini, Pusa Sadabahar and Pusa Ruby | Indian Agricultural Research Institute (IARI), New Delhi, India |
|  | Hisar Arun | CCS, Haryana Agricultural University (CCSHAU), Hisar, India |
|  | IIHR-2274 and IIHR-2201 | Indian Institute of Horticultural Research (IIHR), Bengaluru, India |
|  |  |  |
|  | | |
| **b. Primers for expression analysis** | | |
| **S.No.** | **Gene ID** | **Sequence** |
| 1 | Solyc01g079530 F | ACGGCTCCACCTCCTAAGAA |
|  | Solyc01g079530 R | GACTCTACTCTTTCCCTTCCG |
| 2 | Solyc01g095140 F | CCACTCTCACATAGCGGCGAGA |
|  | Solyc01g095140 R | GCGCGCTAGTTTCAGATCTCTT |
| 3 | Solyc03g020030 F | CTCTATGGTCACGATGGATCT |
|  | Solyc03g020030 R | GCTCCAATGCCCACATTACATT |
| 4 | Solyc05g051200-F | AGGGATTCAACGCGTAATGGA |
|  | Solyc05g051200-R | AGAGACCAAGGACCCCTCAT |
| 5 | Solyc05g052670 F | CCGTCTCCAGCCGAAGCTAA |
|  | Solyc05g052670 R | TCAATTGCTCAGCGCACCAG |
| 6 | Solyc07g008103 F | TTCAACAAATGGCCCAACAAA |
|  | Solyc07g008103 R | GGCTGAGGAGCGGGAGAGGCGG |
| 8 | Solyc07g053740-F | TGCCAGAGAGTTTCGTGGAC |
|  | Solyc07g053740-R | CGTTTCTCCGCTGGATGACT |
| 9 | Solyc07g056570 F | CCGACCCACGAGTCCAGATT |
|  | Solyc07g056570 R | GAACGGCGTGAACCATACCG |
| 10 | Solyc09g014280 F | GGCGGTCAATTGCCGACATC |
|  | Solyc09g014280 R | TGACATCACCATGCGCCACT |
| 11 | Solyc10g009110-F | ACGCGCCGGAGTTATAAAGT |
|  | Solyc10g009110-R | GCGGTGGTTGATTTCACGTC |
| 12 | Solyc10g081570 F | GGACGCATTGACACGCGGACAA |
|  | Solyc10g081570 R | TGCTCCTTCATATCATCAGAC |
| 13 | Solyc12g013700 F | GGTTCAGATGGTGGGGTGAA |
|  | Solyc12g013700 R | TGTGGGAAAGGGTGCAAATG |
| 14 | Solyc12g042500 F | GTGCAGAGTGTCATTGTGTTC |
|  | Solyc12g042500 R | AGGATTTGTGCCTCTATATTTG |
| 15 | Solyc03g078400-F (Actin) | TGTTGCTGTTTTGGATGTGGT |
|  | Solyc03g078400-R | CCTCATCCGAGGGTAATCTGC |
| 16 | Solyc08g062960-F | TTGAGGCGGTCTCTAGTAGC |
|  | Solyc08g062960-R | ACAGGACATTCCTCCAGACT |
| 17 | Soyc09g009100-F | TATTCCCTCTCCTTGGTCTG |
|  | Soyc09g009100-R | TGCCATCAAATATTCACAGTC |
| 18 | Solyc09g065660-F | AGTTCCTTTGTTACATCTCTGC |
|  | Solyc09g065660-R | CCATAAAACTTGATCAGGATCTGC |
| 19 | Solyc06g076570-F | TGCTAAACACTCCTCCAATGA |
|  | Solyc06g076570-R | TGCTTGCTTGCTTTCGATT |
| 20 | Solyc09g011710-F | GCAGGAACCATCACCATATT |
|  | Solyc09g011710-F | GTGGAGTAGTACCACCAAAT |
| 21 | Hsp90-F | TCCTTTCTGTTGAATACTGTGTTG |
|  | Hsp90-R | ATGTTCCATTAATTTGTACGATCC |
| 22 | APX-F | TGATTGAAGAATATTGTATGGC |
|  | APX-R | GCAACAAACATAAAGACACAA |
| 23 | GUS-F | CGGCATCCGGTCAGTGGCAGT |
|  | GUS-R | GCGTGGTCGTGCACCATCAGCAC |
| 25 | NPT-II-F | GCACGCAGGTTCTCCGGCG |
|  | NPT-II-R | CCCGACAGGTCGGTCTTGACA |
| **c. Cloning primers for VIGS** | | |
| 1 | Solyc09g014280 VIGS F | CGGAATTCACCCTGATCATCATACA |
|  | Solyc09g014280 VIGS R | CGGGATCCTCCGGCATCGTTACA |
| 2 | Solyc07g056570 VIGS F | CGGAATTCCACCCTTTGCCGAAAACA |
|  | Solyc07g056570 VIGS R | CGGGATCCTGAACGGCGTGAACCA |
| 3 | Solyc03g020030 VIGS F | CGGAATTCGGGTTGCTACTATTACGGT |
|  | Solyc03g020030 VIGS R | CGGGATCCAAGATCCATCGTGACCA |
|  |  |  |
| **d. Primers for transient overexpression** | | |
| 1 | Solyc03g020030 PBI121F | GCTCTAGAATGGCTCTTCACAAAG |
|  | Solyc03g020030 PBI121R | TCCCCCCGGGTTAATGAGGTTTAACACC |
| 2 | Solyc07g056570 PBI121F | GCTCTAGAATGGCAACTACTACTTC |
|  | Solyc07g056570 PBI121R | TCCCCCCGGGTCATGCCTGATTTGCC |
| 3 | Solyc05g051200 PBI121F | GCTCTAGAATGTCAAGCCCACTAG |
|  | Solyc05g051200 PBI121R | TCCCCCCGGGCTATGATGAAGTCATT |
| 4 | Solyc07g053740 PBI121F | GCTCTAGAATGGCTGTGAAAGAT |
|  | Solyc07g053740 PBI121R | CGGGATCCTTAAACTTCCATAGGT |
| 5 | Solyc10g009110 PBI121F | GCTCTAGAATGGCGCCTAAGGAA |
|  | Solyc10g009110 PBI121R | TCCCCCCGGGTCACATGTTTTCCGG |
| **e. Cloning primers for promoters** | | |
| 1 | Solyc09g014280-FP | ACTAAGCTTGCAAGATTAAAGGGTCATC |
|  | Solyc09g014280-RP | ACTGGATCCGGCATATTTGCAGGGAGTT |
| 2 | Solyc7g056570-FP | ACTCTAGAAGAATATTCCACTAGTCAAC |
|  | Solyc7g056570-RP | ACTGGATCCAGTTGCCATAGCTACCTATT |
